# Premature aging and cancer development in transgenic mice lacking functional CYLD

**DOI:** 10.18632/aging.101732

**Published:** 2019-01-10

**Authors:** Josefa P. Alameda, Ángel Ramírez, Rosa A. García-Fernández, Manuel Navarro, Angustias Page, José C. Segovia, Rebeca Sanchez, Cristian Suárez-Cabrera, Jesús M. Paramio, Ana Bravo, M. Jesús Fernández-Aceñero, M. Llanos Casanova

**Affiliations:** 1 Molecular Oncology Unit, Centro de Investigaciones Energéticas, Medioambientales y Tecnológicas (CIEMAT)/CIBERONC, 28040 Madrid, Spain; 2 Biomedical Research Institute I+12, 12 de Octubre University Hospital, 28040 Madrid, Spain; 3 Department of Animal Medicine and Surgery, Faculty of Veterinary, UCM, 28040 Madrid, Spain; 4 Division of Hematopoietic Innovative Therapies, CIEMAT/CIBERER/II-FJD, 28040 Madrid, Spain; 5 Department of Anatomy, Animal Production and Veterinary Clinical Sciences, Faculty of Veterinary Medicine, University of Santiago de Compostela, Lugo, Spain; 6 Servicio de Anatomía Patológica Hospital Clínico San Carlos, Departamento de Anatomía Patológica, Facultad de Medicina, UCM, Instituto de Investigación Sanitaria del Hospital Clínico San Carlos (IdISSC), 28040 Madrid, España

**Keywords:** CYLD, premature aging, tumor suppressor, NF-κB, keratinocyte differentiation, skin, inflammation

## Abstract

CYLD is a deubiquitinating enzyme known for its role as a tumor suppressor whose mutation leads to skin appendages tumors and other cancers. In this manuscript we report that the tumor suppressor CYLD, similarly to other renowned tumor suppressor genes, protects from premature aging and cancer. We have generated transgenic mice expressing the mutant CYLD^C/S^ protein, lacking its deubiquitinase function, under the control of the keratin 5 promoter, the K5-CYLD^C/S^ mice. These mice express the transgene in different organs, including those considered to be more susceptible to aging, such as skin and thymus. Our results show that K5-CYLD^C/S^ mice exhibit epidermal, hair follicle, and sebaceous gland alterations; and, importantly, they show signs of premature aging from an early age. Typically, 3-month-old K5-CYLD^C/S^ mice exhibit a phenotype characterized by alopecia and kyphosis, and, the histological examination reveals that transgenic mice show signs of accelerated aging in numerous organs such as skin, thymus, pancreas, liver and lung. Additionally, they spontaneously develop tumors of diverse origin. Over-activation of the NF-κB pathway, along with hyperactivation of Akt, JNK and c-Myc, and chronic inflammation, appear as the mechanisms responsible for the premature aging of the K5-CYLD^C/S^ mice.

## Introduction

The *CYLD* gene [1,2] encodes an enzyme (CYLD) that is ubiquitously expressed and contains a deubiquitinating (DUB) domain at the C-terminus, which removes lysine-63 linked polyubiquitin chains. The first function described for CYLD was the inhibition of the nuclear factor (NF)-κB pathway [1], and mutations that inactivate the carboxyl-terminal deubiquitinating domain of CYLD deregulate the NF-κB activity, leading to the development of skin appendages tumors in patients of familial cylindromatosis [2].

The ubiquitous NF-κB family of transcription factors is composed of dimers of five members, being the predominant dimer in skin p65/p50 [3]. In resting cells NF-κB is maintained inactive and its activation by pro-inflammatory signals (such as cytokines IL-1β and TNF-α in the canonical pathway), results in the phosphorylation and posterior degradation of the inhibitor of NF-κB, IκB, enabling a rapid nuclear entry of the NF-κB dimers, and the consequent activation of specific target genes [3]. NF-κB plays a crucial role in various biological processes, such as immune response and inflammation, and its dysregulated activity leads to the development of various autoimmune disorders, as well as to cancer development [4].

The skin is composed of three layers: epidermis, dermis and hypodermis; and also contains specialized structures, such as hair follicles (HF) and sebaceous and sweat glands. The balance between cell proliferation and differentiation of the epidermis must be maintained in order to preserve their functionality. The HF is a highly sensitive appendage undergoing continuous regeneration throughout life: HFs undergo periodic phases of rapid growth (anagen), apoptosis-driven regression (catagen) and relative quiescence (telogen). HF characteristics associated with each phase are morphologically distinct and distinguishable [5]. Loss of homeostasis of the epidermis and skin appendages leads to numerous skin alterations, such as alopecia, inflammatory diseases and non-melanoma skin cancer (NMSC). Our group and others have described that CYLD acts as a suppressor of the development and progression of the most aggressive form of the NMSC, i.e. skin squamous cell carcinomas (SCC) [6–9]. However, the role that CYLD plays *in vivo* in the epidermis and HF homeostasis has not been fully characterized.

In recent years, several genetic studies have associated the loss of CYLD functionality with the dysregulation of NF-κB, JNK, c-Myc or Akt [10–12] and the development of different types of cancers of high prevalence in the population (multiple myeloma, hepatocarcinomas, lung, breast and gastric cancers, etc.) [13]. Therefore, it seems that CYLD, like other well-known tumor suppressor proteins such as Ink4a, Arf and PTEN, acts as a tumor suppressor in a variety of malignancies. It is interesting that these other renowned tumor suppressors develop important additional roles protecting from aging [14–16]. However, the possible role of CYLD as an aging guard has not been yet investigated.

The dual role of the tumor suppressors protecting from cancer and aging is not surprising, as it has been considered that age is the most significant risk factor for cancer development [17]. It is remarkable that it is widely accepted that the activation of the NF-κB signaling pathway is the driver of aging [18], since the genetic or pharmacological inhibition of NF-κB results in the blocking of aging and even the reversion of tissue characteristics of old mice to those of young mice [19,20]. Thus, the continuous NF-κB hyperactivation has been directly linked to the aging process [4,20]; moreover, abnormal NF-κB activation is known to occur in diverse age-associated diseases (diabetes, osteoporosis, neurodegeneration etc.) [4].

It is relevant to mention that although there are many other molecules whose activation have been implicated in pro-aging and longevity processes, such as c-Myc, Akt and JNK, all of them converge in the activation or inhibition of NF-κB signaling pathways respectively [4]. Also, chronic inflammation has been considered as a predominant and recurrent factor that is associated with the process of physiological and pathological aging, and, in this case, NF-κB is also found on the axis of the inflammatory network of aging, since it is activated by innate/inflammatory responses, provoking a host defense mechanism, responsible for the release of SASP (senescence-associated secretory phenotype) molecules, principally IL-6 and TNF-α, which in turn favors the aging process and leads to the activation of many pro-inflammatory signaling pathways, mainly the NF-κB pathway [21,22].

We reasoned that CYLD, a tumor suppressor that is an inhibitor of NF-κB activation, and consequently of inflammation, could also play an important role against aging. To study this possibility, we have generated a new model of transgenic mice, the K5-CYLD^C/S^ mice, carrying the mutant CYLD^C/S^ construct [6,9,23] under the regulatory elements of the keratin 5 (K5). These mice express a mutant CYLD^C/S^ protein defective in its DUB function in the skin and other numerous organs, and our results show that they exhibit signs of accelerated aging from very early ages; they also exhibit inflammation and develop spontaneous tumors in many organs. Therefore, our data indicate that CYLD, like other well-known tumor suppressor genes, also acts as an aging protector. Moreover, we propose that this is an important mechanism through which CYLD exerts its function as a tumor suppressor.

## RESULTS

### K5-CYLD^C/S^ transgenic mice have an impaired deubiquitination function

**Figure 1:**
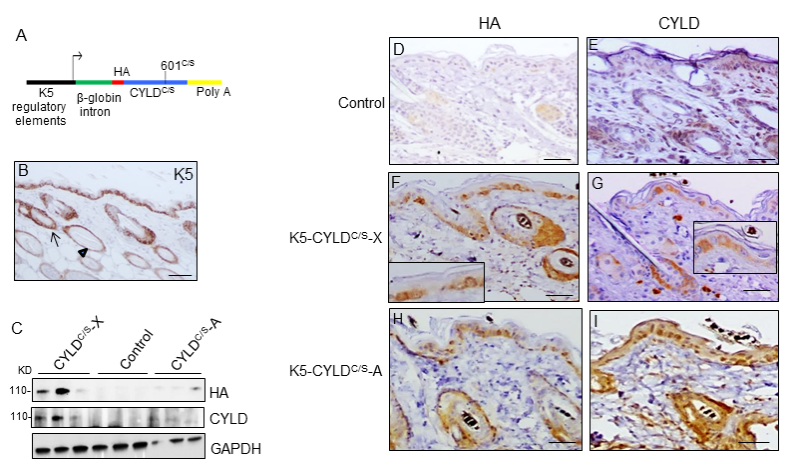
**Analysis of the expression of the endogenous and the mutant CYLD protein in the K5-CYLD^C/S^ mice.** (**A**) Scheme of the construction used to obtain the K5-CYLD^C/S^ mice. (**B**) Representative image showing the expression of K5 in the back skin of Control mice. Arrow: sebaceous gland; arrow head: ORS. (**C**) Analysis by WB of the expression of HA and CYLD in total protein extracts from the back skin of 30 day-old control and transgenic mice. Both lines of K5-CYLD^C/S^ mice express higher levels of CYLD than Controls. HA was not detected in Control mice. GAPDH was used as a loading control. Immunostaining -with HA (**D, F, H**) and CYLD (**E, G, I**) antibodies- of back skin samples from Control (**D, E**) and transgenic (K5-CYLD^C/S^-X and K5-CYLD^C/S^-A) mice (**F-G, H-I,** respectively). HA is not expressed in Control mice (**D**). In the K5-CYLD^C/S^ mice both, the expression of HA (**F, H)** and CYLD **(G, I)** follows the expression pattern of K5. Scale bars: 250μm (B); 150μm (D-I).

We have generated transgenic mice expressing a mutant CYLD protein (defective in the DUB function) under the control of the K5 promoter. The transgenic construct contains a mutant CYLD^C/S^ complementary DNA carrying a 601C/S point mutation in the cysteine box of the DUB domain (Fig. 1A), resulting in a catalytically inactive protein that acts as a dominant negative protein and is able to compete with the endogenous CYLD [2,6,9,23]. The K5 derived sequences included in this construct drive transgene expression to the skin, specially to keratinocytes of the basal layer of the epidermis, the outer root sheath (ORS) of hair follicles, and the mitotically active basal cells of the secretory units that are the source of new secretory sebaceous glands [24] (Fig. 1B). K5 promoter also drives transgenic expression to other organs besides skin, i.e. tongue, palate, thymus, lung, stomach etc [24]. Two independent lines of transgenic mice were obtained, named as K5-CYLD^C/S^-A and K5-CYLD^C/S^-X. We verified by immunoblotting using two different antibodies (against CYLD or against the HA tag contained in the construct), that both lines expressed the transgene, although at different levels (Fig. 1C). The expression of the transgenic protein was also detected *in situ,* by immunohistochemical staining in different tissues (such as back and tail skin and tongue) of both lines of transgenic mice, and we found that it was expressed following the K5 expression pattern (Fig. 1, F-I; Fig. S1). We also checked in both lines of transgenic mice, that, as expected, the CYLD^C/S^ mutant was catalytically inactive and inhibited the DUB function of the endogenous CYLD, as we previously described that occurred in the epidermal HaCaT-CYLD^C/S^ and PDVC57-CYLD^C/S^ cells [6,9] (Fig. S2). The following analyses were performed in both lines of transgenic mice and obtained similar results.

### CYLD controls hair follicle growth cycle

**Figure 2:**
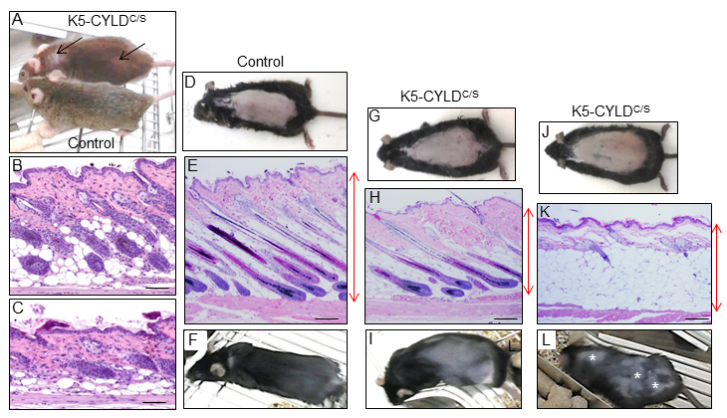
**Analysis of the hair regrowth in Control and K5-CYLD^C/S^ mice**. (**A**) Representative image of the patches of diffuse alopecia (arrows) of the transgenic mice (7-month-old mice are shown). (**B, C**) Representative images of the skin of 1-month-old mice. (**D-L**) Representative images of a hair growth experiment. The back of 7-week-old mice was shaved and the skin was collected 16 days after depilation. (**D-F**) Hair regrowth in Control mice. (**G-L**) Hair regrowth in the K5-CYLD^C/S^ mice. (**D, G, J**) Representative images of freshly depilated mice of the corresponding phenotypes (day 0 of the experiment). (**E, F**) 16 days after shaving Control HFs were in the anagen-catagen I phase (**E**) and mice exhibited a homogeneous hair regrowth (**F**). (**H**) Histology showing initiation of the anagen phase of the hair cycle 16 days after shaving the back of a transgenic mouse. The macroscopic view of this mouse showed that hair was very short and hardly visible (**I**). (**K**) Histology of a section of the back skin of a K5-CYLD^C/S^ mouse showing a delay in the growth of the new hair, so that 16 days after shaving it still remains in the telogen phase. (**L**) Macroscopically these areas correspond to those lacking hair in the back skin of the transgenic mouse (white asterisks). Red arrows show differences in the thickness of the skin between Control (**E**) and transgenic mice (**H, K**). Scale bars: 280 μm.

Young transgenic mice appeared normal and healthy; however, they showed an abnormal, untidy hair and patches of diffuse alopecia (Fig. 2A), visible from early age, i.e. 3-month-old mice. In addition, the histology of skin sections showed that while hair follicles in dorsal skin of 1-month-old Control mice had progressed to the anagen phase of the second hair growth cycle (Fig. 2B), as it occurs in normal mice at this age, HF in dorsal skin of 1-month-old transgenic mice remained in the first telogen phase (Fig. 2C). To ascertain whether CYLD had a role in hair follicle homeostasis and/or hair cycling, we depilated the dorsal skin of 7-week-old mice (depilation allows the study of HF cycle in conditions of fully synchronized anagen [25]). Sixteen days later Control mice exhibited a homogenous short-haired layer (Fig. 2F), corresponding histologically to HF in the anagen-catagen I phase of the hair growth (Fig. 2E), while HFs of K5-CYLD^C/S^ mice had just entered the anagen phase (Fig. 2H) or still remained in the telogen phase (Fig. 2K) and, accordingly, hair was not visible yet or it had grown in a patched form (Fig. 2I and L). These histological differences were also reflected in the decrease in the skin thickness of the transgenic mice. Therefore, these findings indicate that CYLD is a positive stimulator of hair growth and that the DUB function of CYLD is essential for HF anagen induction of the second hair cycle.

### K5-CYLD^C/S^ transgenic mice exhibit a premature aging skin phenotype

**Figure 3:**
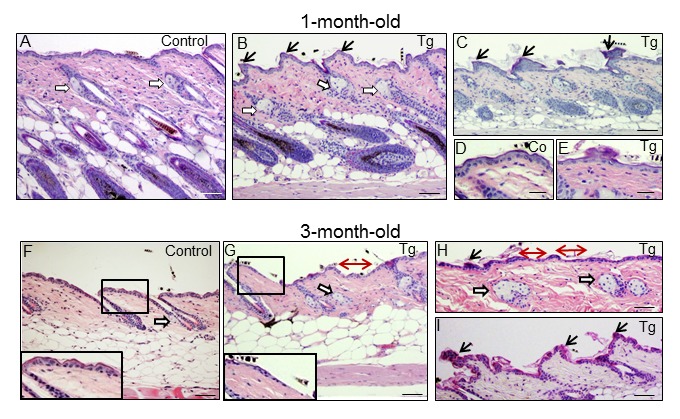
**Histological alterations in the back skin of 1 and 3-month-old K5-CYLD^C/S^ mice.** (**A-E**) Histology of the back skin of 1-month-old Control (**A, D**) and K5-CYLD^C/S^ mice (**B, C, E**). (**A**) Observe small sebaceous glands (white arrows) and HFs in the anagen phase in Control mice. (**B, C**) Note in transgenic mice the presence of moderately hyperplastic sebaceous glands, epidermal ridges and HFs initiating the anagen phase of the second hair growth cycle. (**D, E**) Slight thinning of the epidermis of K5-CYLD^C/S^ mice. (**F-I**) Histology of the back skin of 3-month-old Control (**F**) and K5-CYLD^C/S^ mice (**G-I**). (**F**) Note small sebaceous glands and telogenic HFs in Control mice. (**G-I**) Observe marked epidermal atrophy; abundant epidermal ridges of pyknotic keratinocytes, increased hyperplasia of the sebaceous glands and areas with orphan sebaceous glands lacking hair follicles in transgenic mice. White arrows: sebaceous glands; black arrows: epidermal ridges of pyknotic keratinocytes; double-headed red arrows: areas of epidermal atrophy. Scale bars: 250 μm (**C, F-H**); 200 μm (**A, B**); 180 μm (**I**); 150 μm (**D, E**).

**Figure 4:**
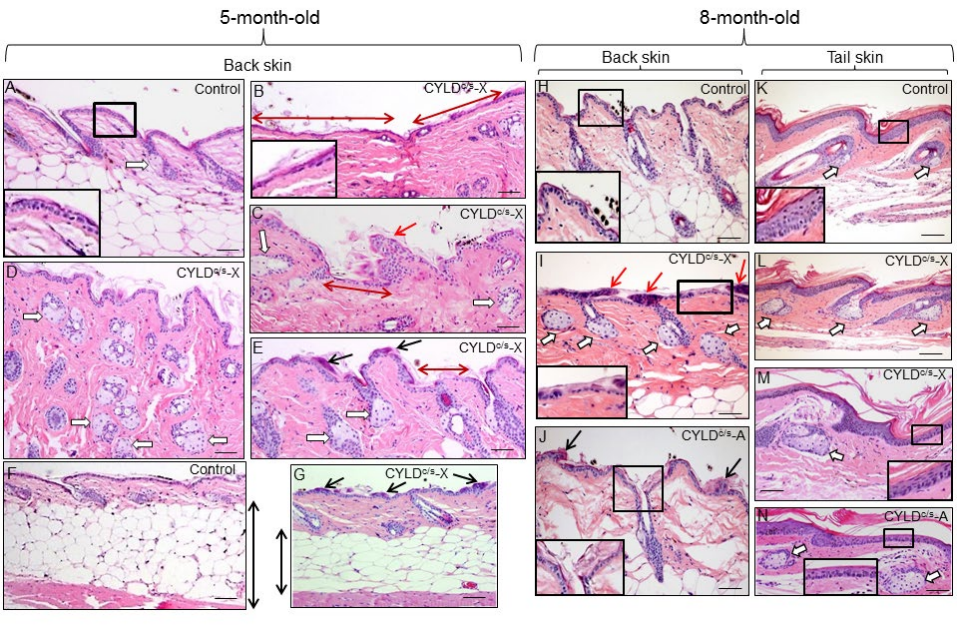
**Histopathological signs of premature aging in the back skin of young (5- and 8-month-old) transgenic mice**. Representative histology of the back skin of 5-month-old Control and transgenic mice (**A-G**). (**A**) Histology of the back skin of a Control mouse. Observe the presence of small sebaceous glands (white arrow) and 3 layers of keratinocytes in the interfollicular epidermis (higher magnification is showed in the inset). (**B-E**) The epidermis of the K5-CYLD^C/S^ mice shows frequent and extensive areas of atrophy (double-headed red arrows in **B** and **C**; also compare the inset in **B** with that of **A**); as well as papillomatous hyperplasia (red arrow in **C**) and epidermal ridges (black arrows in **E** and **G**). Abundant hyperplastic sebaceous glands -often orphan, were detected (white arrows in **C-E**). (**F-G**) Observe the scarce adipose tissue present in the skin of the transgenic mice (compare the length of the double-headed black arrows). Representative histology of the back skin of Control (**H**) and transgenic mice (**I, J**) of 8-month-old. Observe in the K5-CYLD^C/S^ mice the presence of papillomatous hyperplasia (red arrows in **I**); epidermal ridges (black arrows in **J**); abundant hyperplastic sebaceous glands (white arrows), some of them orphan (without HF) (**I**), and patchy epidermal atrophy associated to moderate hyperkeratosis (compare the inset in **H** with those of **I** and **J**). Representative histological images of the tail skin of Control (**K**) and transgenic mice (**L-N**). Note in the skin of transgenic mice the presence of hyperplastic sebaceous glands, most of them orphan (white arrows), and epidermal atrophy (compare the insets in **K** with those of **M** and **N**). Images of the histology of both K5-CYLD^C/S^-X and K5-CYLD^C/S^-A are shown. White arrows, sebaceous glands; black arrows: epidermal ridges; double-headed red arrows: areas of epidermal atrophy. Scale bars: 150 μm (**C, E**); 180 μm (**A, B, D, I, J, L-N**); 200 μm (**H, K**) and (**F, G**) 350 μm.

**Figure 5:**
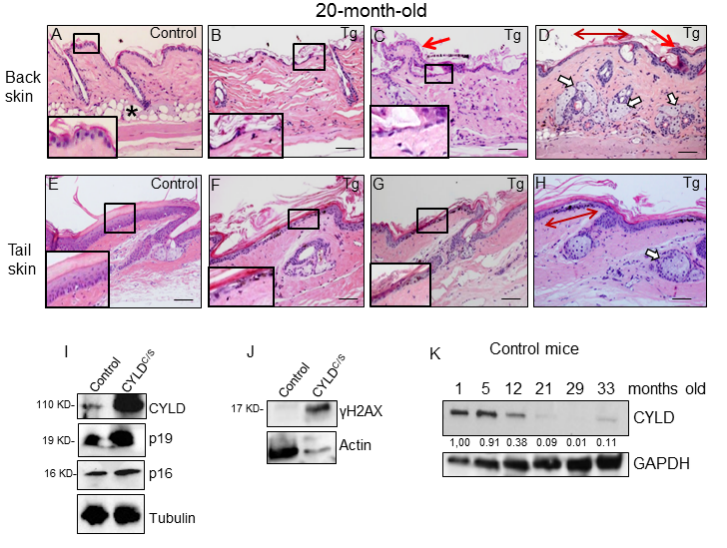
**Histological and molecular signs of premature aging in the back skin of transgenic mice.** (**A-H**) Representative histological images showing the back skin of 20-month-old Control mice (**A**) and the severe aging phenotype of the back skin of 20-month-old transgenic mice (**B-D**). (**B-D**) Note severe epidermal atrophy (compare insets in **A** with those of **B** and **C**; double-headed red arrow in **D**); foci of papillomatous hyperplasia (red arrows in **C** and **D**); numerous hyperplastic sebaceous glands, most of them orphan and grouped in the dermis (**D**); reduced number of HFs, and scarce or even lack of adipose tissue (compare **A** with **B-D**) in the back skin of the K5-CYLD^C/S^ mice. (**E-H**) Tail skin of Control (**E**) and transgenic (**F-H**) mice. Note the presence of hyperplastic sebaceous glands and extensive epidermal atrophy (compare inset in **E** with those in **F** and **G**) in the tail of the K5-CYLD^C/S^ mice. (**I, J**) WB of total protein extracts from skin of 12-month-old (**I**) and 6-month-old (**J**) showing elevated levels of p16, p19 and γH2AX in the K5-CYLD^C/S^ mice. Tubulin and Actin are used as control loading. (**K**) WB of total protein extracts from the skin of Control mice from 1 to 33 months of age showing the decreased expression of CYLD as mice age. GAPDH is used as a control loading. White arrows: sebaceous glands; red arrows: papillomatous hyperplasia; double-headed red arrows: areas of epidermal atrophy. Scale bars: 250 μm (**A-D**); 200 μm (**E-H**).

In addition to diffuse alopecia, young transgenic mice (8-12 months old, and even younger) also showed an abnormal posture, characterized by excessive outward curvature of the spine (kyphosis) (Fig. S3). These features, alopecia and kyphosis in youth, are suggestive of premature aging. To check for the presence of other signs of accelerated aging in the K5-CYLD^C/S^ mice, histological analyses of the skins of mice at different ages were performed, as this organ is one of the first in which the signs of aging manifest (number and age of analyzed mice is showed, Table S1). We observed that at 1 month of age, in addition to the delay in the entry into the second hair growth cycle commented above (Fig. 2C), K5-CYLD^C/S^ mice presented a mild hyperplasia of the sebaceous glands, showing 6-8 mature sebaceous cells (Fig. 3B), versus 3-4 mature sebaceous cells in Control mice (Fig. 3A). Additionally, the interfollicular epidermis tended to be slightly thinner in the transgenic mice (compare Fig. 3D and E), and showed regions of thicker epidermis forming ridges of pyknotic keratinocytes (Fig. 3B and C). At 3 months of age, the skin phenotype of K5-CYLD^C/S^ mice was more obvious, showing a further pronounced thinning of the interfollicular epidermis, which presented areas with just one layer of flat keratinocytes, versus the 3 layers usually found in Control mice (compare insets in Fig. 3F and G); the epidermal ridges were more abundant (Fig. 3H and I), and orphan and hyperplasic sebaceous glands, with absence of HF were frequently observed (Fig. 3G and H), being macroscopically coincident with the incipient alopecia observed in the scalp of 3-month-old transgenic mice (not shown). Afterwards, additional young mice of 5- and 8-month-old were analyzed (Fig. 4 A-N; images of both K5-CYLD^C/S^-X and -A mice are shown) and we found that the skin alterations of the transgenic mice were quickly emphasized as the mice grew; thus, 5-month-old K5-CYLD^C/S^ mice exhibited a very prominent phenotype, showing large areas of atrophic interfollicular epidermis in the back skin (Fig. 4B,C,E,I,J) alternating with regions of large epidermal ridges (Fig. 4 E, G) which often form foci of papillomatous hyperplasia, giving the appearance of a thin and wrinkled skin (Fig. 4 C, I, J). Also, transgenic mice showed scarcity of adipose tissue in the hypodermis (compare Fig. 4 F, G), which together with the atrophic epidermis contributes to the thinning of the skin in these mice. In addition, these animals showed an important reduction in the number of hair follicles in the back skin (Fig. 4 B, C, J), as well as abundant hyperplastic and orphan sebaceous glands clustered in the dermis (Fig. 4 C-E; I, L-N). The analysis of the tail skin of the K5-CYLD^C/S^ mice revealed histological alterations similar to those in the back skin, i.e., hyperplasia of sebaceous glands, and thinning of the interfollicular epidermis (Fig. 4 K-N). In addition to these symptoms, the back skin of older transgenic mice (20-month-old) showed a striking lack of adipose tissue and the presence of profuse orphan sebaceous glands (Fig. 5 B-D) which resulted in the development of a severe alopecia of the K5-CYLD^C/S^ mice. Also, aggravation of the phenotypic alterations of the tail skin was detected in the 20-month-old transgenic mice (Fig. 5 F-H); however, 20-month-old Control littermates did not show these alterations neither in the back skin nor in the tail skin (Fig. 5).

Besides back and tail skin, other stratified epithelia of the K5-CYLD^C/S^ mice showed relevant alterations suggestive of early aging; it was the case of the palate, tongue and plantar skin; the epithelia of the snout and eyelids; also, the Meibomian glands presented a marked hyperplasia (Fig. S4).

Therefore, the signs of aging found in the skin of the K5-CYLD^C/S^ mice from 1 month of age can be considered characteristics of premature aging, as they were not manifested in the skin of Control mice until they were over 24 or 28 months old, suggesting that CYLD protects from aging. To further reinforce our findings, we analyzed the level of expression of classical molecular markers of aging, i.e., p16 and p19, and found that these were elevated in the skin of the K5-CYLD^C/S^ mice (Fig. 5I); which strengthen our observations about the accelerated aging of the transgenic mice, since the levels of p16INK4a (and, to a lower extent, also p19ARF) increase with aging in almost all tissues analyzed both in mice and humans [26,27]. Moreover, we analyzed levels of γH2AX, a molecular marker of DSBs, whose elevation has been proposed as a molecular marker of aging [28], and found that levels of γH2AX were also increased in the skin of the K5-CYLD^C/S^ mice (Fig. 5J).

Additionally, we have analyzed the level of expression of CYLD in the skin of Control mice at different ages and found an important decrease in the amount of CYLD protein with aging, i.e. there was a diminished expression of CYLD in the skin of 12 month-old mice respect to that observed in younger mice (1-month-old); and, in aged mice (from 21-month-old), the expression of CYLD was hardly detected, being observed a decrease of more than 10 fold in the expression levels of CYLD (Fig. 5K). Therefore, all these results support the role of CYLD as a suppressor of aging in the skin.

### Impaired differentiation of the epidermis of K5-CYLD^C/S^ mice

**Figure 6:**
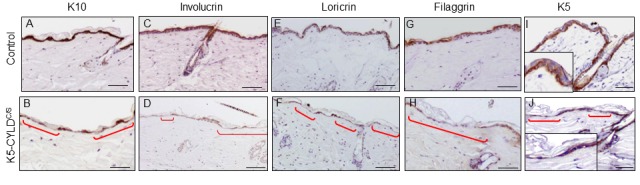
**Deficient differentiation in the skin of K5-CYLD^C/S^ mice.** Representative immunostainings of the back skin of 20-month-old mice (**A-J**). Observe the strong expression of the epidermal differentiation proteins Involucrin, Loricrin and Filaggrin in the suprabasal layers of the epidermis of Control mice (**A,C,E,G**); and the weak and discontinuous expression of these proteins in the epidermis of K5-CYLD^C/S^ mice (**B,D,F,H**), specially faint in the areas of epidermal atrophy (red brackets). (**I, J**) Representative images corresponding to the immunostaining of the back skin of Control (**I**) and transgenic (**J**) mice with the K5 specific antibody. (**I**) Strong K5 staining in basal keratinocytes of Control mice. A faint and patched expression is detected in the epidermis of the transgenic mice, especially in the regions of atrophic epidermis (indicated by red brackets). Scale bars: 180 μm (**A-H**); 150 μm (**I, J**).

The analysis of the proliferation rate in the skin showed increased levels of BrdU and Ki67 staining in the sebaceous glands of K5-CYLD^C/S^ mice (Fig. S5; S6 and S7), which is in accordance with its hyperplastic condition. No differences in apoptosis were found (measured by Caspase 3 cleaved immunostaining; data not shown). We then analyzed whether the differentiation of the epidermis of the K5-CYLD^C/S^ mice was affected by the lack of the DUB function of CYLD. The immunohistochemical examination of the early (K10, involucrin) and late (loricrin and filaggrin) differentiation markers showed a continuous and strong staining for all these markers in the suprabasal layers of the epidermis of Control mice; by contrast, the skin of K5-CYLD^C/S^ mice showed a pattern of discontinuous and scarce staining (Fig. 6 A-H). This deficiency in the epidermal differentiation of the skin of transgenic mice was also confirmed by a semiquantitative analysis of the expression of these differentiation markers (Fig. S8). In addition, K5 staining of the basal layer of the epidermis of transgenic mice was also abnormal, showing areas of positive staining containing flat keratinocytes, with nuclei parallel to the basal membrane, alternating with no-staining skin, in which almost no keratinocytes were detected (Fig. 6J). This K5 staining pattern contrasted greatly with the continuous K5 expression found in the basal layer of the epidermis of Control littermates (Fig. 6I). Therefore, our results suggest that the atrophic skin of the transgenic mice is linked to alterations in the morphology of the basal keratinocytes and the impairment of the early and terminal epidermal differentiation.

### The skin of K5-CYLD^C/S^ transgenic mice displays chronic activation of NF-κB and other pro-aging pathways, along with increased inflammation

**Figure 7:**
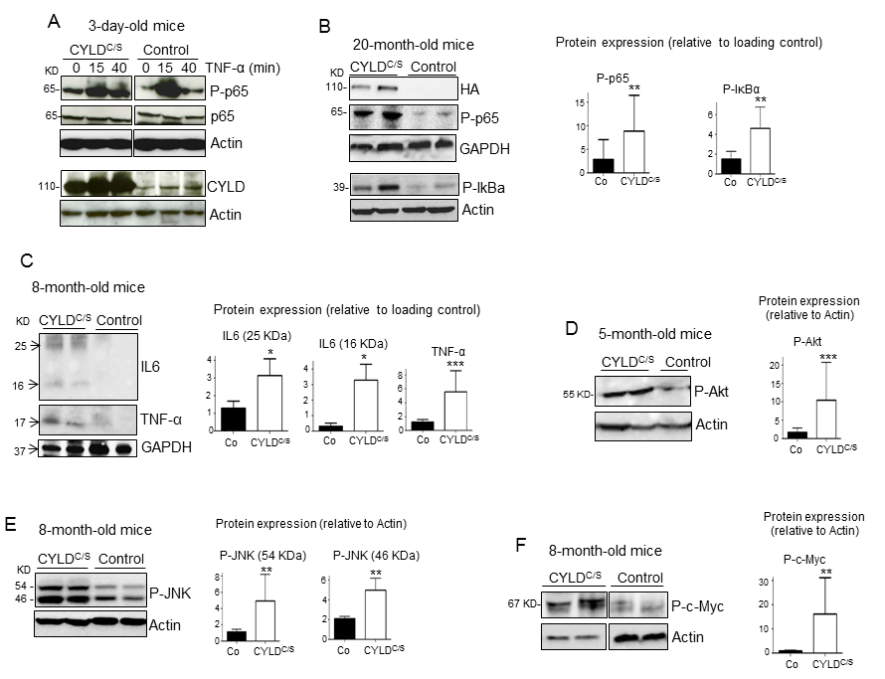
**Overactivation of the NF-κB, and other pro-aging pathways, along with increased IL6 and TNF-α expression in the skin of the K5-CYLD^C/S^ mice**. (**A**) p65 and IκBα phosphorylation kinetics in the back skin of 3-day-old Control and transgenic mice treated with TNF-α for the indicated times. (**B**) WB showing over-activation of the classical NF-κB signaling pathway (P-p65 and P-IκBα) in the skin of 20-month-old transgenic mice. (**C**) Increased expression of the inflammatory cytokines TNF-α and IL6 in the skin of the K5-CYLD^C/S^ mice. (**D-F**) WB showing the hyperactivation (phosphorylation) of Akt (**D**), JNK (**E**) and c-Myc (**F**) in the skin of adult K5-CYLD^C/S^ mice. Graphic representations of the densitometric analysis of western blots corresponding to extracts from 5-7 animals of each genotype are shown. Mann-Whitney U test was used for statistical analysis. (*p<0.05; **p<0.01; ***p<0.001).

Searching for the mechanisms responsible for the premature aging of the skin of K5-CYLD^C/S^ mice, we first studied the activation of the NF-κB signaling in the skin of 3-day-old mice and found that in the unstimulated, basal state, transgenic mice showed increased activation of NF-κB (measured as P-p65 levels), as well as a long-lasting activation after 40 minutes of treatment with TNF-α (Fig. 7A). To analyze whether the hyperactivation of NF-κB occurred only in newborn mice, coinciding with a rapid skin growth, we analyzed the activation of NF-κB in the skin of older mice (20-month-old), and found that P-p65 was also increased in these transgenic mice (Fig. 7B). In addition, elevated phosphorylation of the inhibitor of NF-κB, IκBα, was detected (Fig. 7B), which may also contribute to the increased levels of P-p65 observed. Thus, our results indicate that the skin of transgenic mice exhibit a constitutive activation of the NF-κB canonical pathway, from birth to advanced age. Since a relevant mechanism through which NF-κB activation promotes aging is by upregulating the expression of inflammatory cytokines, we analyzed by Western blot the expression of IL-6 and TNF-α in skin and found that they were significantly increased in that of transgenic mice (Fig. 7C); in addition, they were also increased in the serum of the K5-CYLD^C/S^ mice, mainly IL6 (Fig. S9). Although NF-κB activation is the main regulator of aging, it has been described that the activation of other pathways, which in turn feeds the NF-κB activation, such as Akt, JNK and c-Myc, also favors aging. In agreement with the reported negative regulation of JNK and c-Myc activation by CYLD [29], WB analysis of these molecules showed increased Akt, JNK and c-Myc activation (measured as levels of P-Akt, P-JNK and P-c-Myc respectively) in the skin of transgenic mice lacking the DUB function (Fig. 7 D-F).

### K5-CYLD^C/S^ transgenic mice exhibit a premature aging of thymus

**Figure 8:**
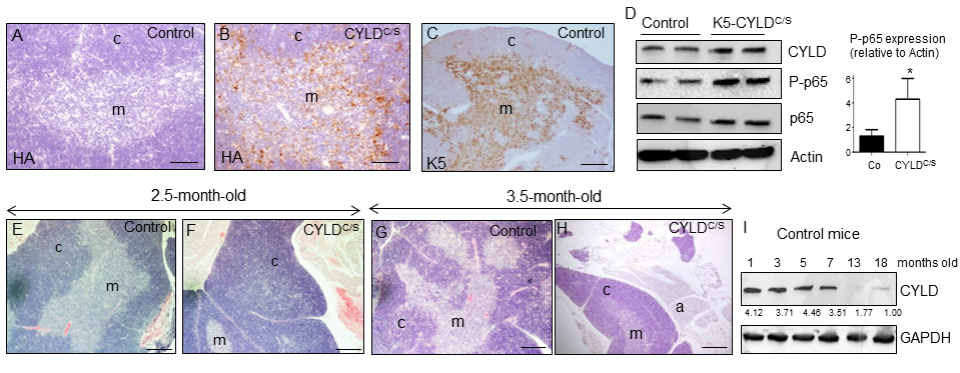
**Premature thymic involution and over-activation of NF-κB in the thymus of K5-CYLD^C/S^ mice.** (**A-C**) Analysis of the expression of the transgene by immunostaining with a specific antibody against the HA tag. Expression of HA is detected in the medulla of the thymus of the K5-CYLD^C/S^ mice (**B**), following the expression pattern of the K5 (**C**), while it is not detected in the Controls (**A**). (**D**) Analysis by WB of the expression of the transgene in protein extracts from isolated thymic cells of mice of 3.5-month-old. Note the overactivation of NF-κB (increased levels of P-p65) in the K5-CYLD^C/S^ mice. Mann-Whitney U test was used for statistical analysis. (*p<0.05). (**E, F**) Histological analysis of the thymus of 2.5-month-old mice. Observe the expansion of the cortical zone and reduction of the medullar region in the thymus of transgenic mice (**F**). (**G, H**) H&E staining of 3.5-month-old Control (**G**) and K5-CYLD^C/S^ mice (**H**) thymus. A representative image of the thymic atrophy and infiltration of white adipose tissue in the thymus of transgenic mice (**H**) is shown. (**I**) Western blot showing the decreased expression of CYLD with age in the thymus of control mice. M, medulla. C, cortex. a, adipose tissue. Scale bars: 200 μm (**A, B**); 300 μm (**C**); 350 μm (**E-H**).

Together with the skin, other organ that is highly susceptible to premature aging is the thymus [19]. As the K5 promoter also drives transgene expression to this organ [24], we first analyzed whether the transgene was expressed in the thymus of the K5-CYLD^C/S^ mice, and found that it was mainly detected in the epithelial cells of the medullar area (Fig. 8B) of the thymus, coincident with the pattern of expression of K5 in this organ (Fig. 8C); WB also confirmed the expression of the transgene in the thymus of the K5-CYLD^C/S^ mice (Fig. 8D). Then, we performed a histological analysis of the thymus at different ages (see Table S2) and found that in very young animals (2.5-months-old) alterations suggestive of premature aging took place. These changes consisted in the expansion of the cortical area, while a reduction of the medullar region occurred (Fig. 8F). Moreover, in 3.5 months-old transgenic mice an involution of the thymus was observed, as reflected by the atrophy of the organ and the infiltration of fat cells at expense of thymic tissue; by contrast, thymic involution was not observed in the thymus of aged-matched Control littermates (Fig. 8G and H), being the involution of the thymus considered as one of the most characteristic changes of the aging immune system [30,31]. Since the NF-κB pathway has been shown to be critically involved in thymic aging [32], we analyzed NF-κB activation by WB and found that it was overactivated in the thymus of the K5-CYLD^C/S^ mice (Fig. 8D), suggesting that, as in the skin, the overactivation of NF-κB in the thymus could explain the accelerated aging of this organ. To further reinforce our findings, we analyzed the level of expression of CYLD in the thymus of Control mice at different ages and found that it decreases with aging, mainly from 7 months of age (Fig. 8I), reinforcing our finding of the role of CYLD as a protector of aging in the thymus.

### K5-CYLD^C/S^ mice exhibit inflammation and other signals of premature aging in further organs

**Figure 9:**
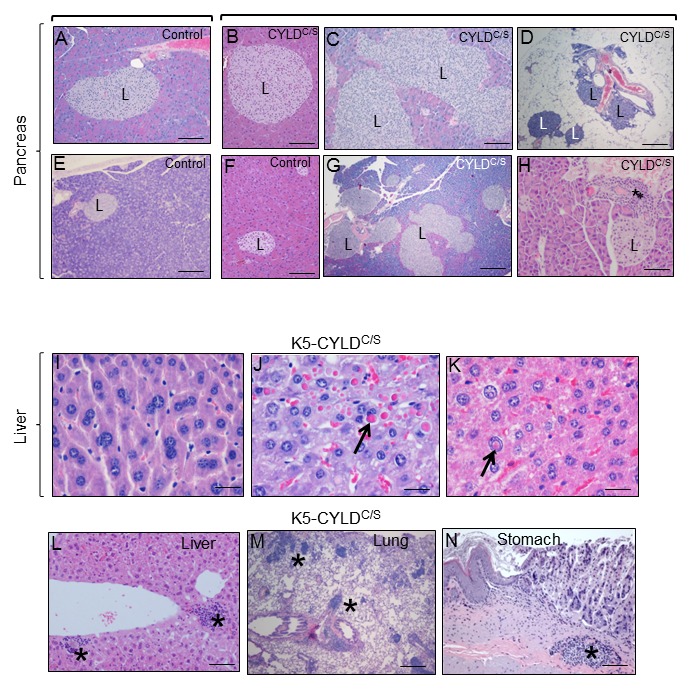
**Alterations found in the pancreas, liver, lung and stomach of the K5-CYLD^C/S^ mice suggestive of early aging of the K5-CYLD^C/S^ mice**. (**A-H**) Histopathologic analysis by H&E staining of pancreas from 5-month-old (**B**, **F**) and 12-month-old Control and transgenic mice. (**A, E, F**) Pancreas from Control mice: note the presence of Islets of Langerhans (L) of heterogeneous but moderate size. (**B-D; G, H**) Histology sections representatives of pancreas from K5-CYLD^C/S^ mice. Note the hyperplasia of the Islets of Langerhans (**B, C, G, H**). (**D**) Extrapancreatic location of the Islets of Langerhans, in the peripancreatic fat, observed in the K5-CYLD^C/S^ mice. (**H**) Foci of inflammation (asterisk) in the pancreas of K5-CYLD^C/S^ mice. (**I-N**) Histopathological analysis of liver, lung and stomach sections from different organs of 20-month-old K5-CYLD^C/S^ mice. (**I-K**) Representative images showing anisokariosis (**I**), eosinophilic intracytoplasmic inclusions (arrow in **J**), intranuclear eosinophilic inclusions (arrow in **K**), and inflammation foci (asterisks in **L**) in the liver. (**M**) Example of inflammation foci observed in the lung. (**N**) Stomach with an inflammation focus. The pancreas of 4 Control and 4 transgenic mice of 5- and 12-month-old were analyzed. Number of animals whose liver, lung and stomach has been analyzed is showed in Table 1. Asterisks: Inflammation. Scale bars: 250 μm (**A, B, D-F**); 350 μm (**C**); 500 μm (**G**); 150 μm (**H; L-N**); 40 μm (**I-K**).

We found that, in addition to skin and thymus, K5-CYLD^C/S^ mice displayed signs of premature aging in other organs. Among them, it was remarkable the accelerated aging of the pancreas, liver, lung and stomach (Fig. 9). Features of early aging of the pancreas in the transgenic mice are the presence of huge islets of Langerhans detected in transgenic mice from 5-month-old (Fig. 9B, C and G), often observed in extrapancreatic locations, i.e. in the peripancreatic fat (Fig. 9D), and the mild chronic inflammation detected even in young mice (since 3-months-old) [33] (Fig. 9H). The liver of transgenic mice also exhibited marked aging-related lesions such as anisokaryosis, anisocytosis, karyomegalia, and inter- and- intranuclear eosinophilic inclusions (Fig. 9I-K) as well as mild to moderate inflammation, i.e., multifocal chronic hepatitis (Fig. 9L). These alterations were not found in the liver of aged-matched Control littermates, with the exception of inflammatory cells that were sometimes detected, although in lower numbers than those found in the liver of transgenic mice (data not shown). Lungs of K5-CYLD^C/S^ mice showed moderate inflammation, consisting in BALT (Broncus Associated Lymphoid Tissues) hyperplasia, which is typically found in lung of elderly mice, but it was observed in young transgenic mice (Fig. 9M); by contrast, Control age-matched littermates showed only a discrete inflammation in the lungs (not shown). Other organ showing evident lymphocyte infiltration was the stomach of K5-CYLD^C/S^ mice (Fig. 9N). Therefore, our results, showing several histological signs of aging in many organs of the transgenic mice, along with the chronic inflammation detected in those organs suggest that K5-CYLD^C/S^ mice undergo a systemic premature aging.

### Aged K5-CYLD^C/S^ transgenic mice develop tumors in many organs

**Figure 10:**
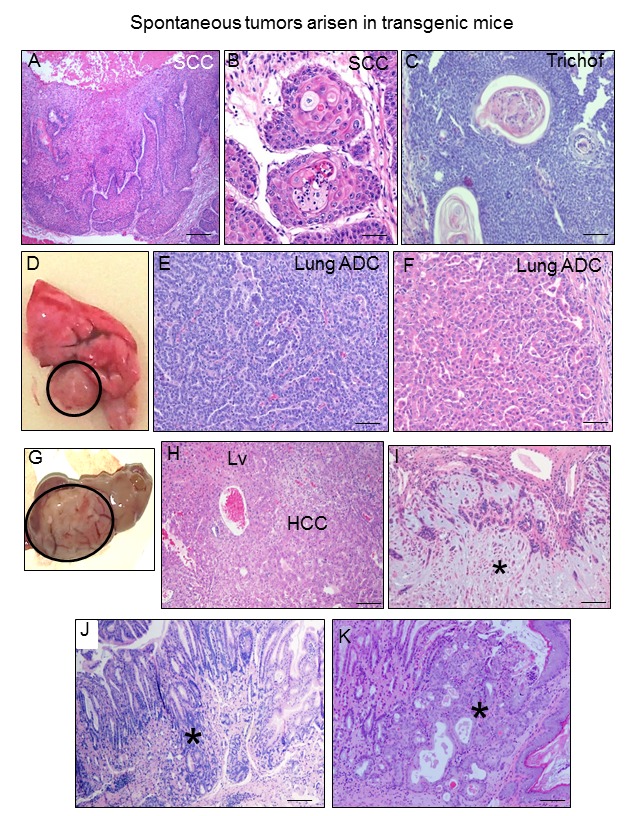
**K5-CYLD^C/S^ mice develop spontaneous tumors in many organs**. (**A-C**) Skin tumors. Infiltrating SCC arisen in the back skin of 8-month-old transgenic mouse (**A, B**). (**C**) Hair follicle derived tumor (trichofolliculoma) developed in the snout of a K5-CYLD^C/S^ mouse. (**D**) Macroscopic appearance of a lung adenocarcinoma. (**E**) Lung acinar adenocarcinoma. (**F**) Lung papillary adenocarcinoma. (**G, H**) Hepatocellular carcinoma (HCC); liver (Lv). (**I**) Mammary adenoepithelioma (asterisk). (**J**) Well differentiated gastric adenocarcinoma (asterisk). (**K**) *In situ* gastric carcinoma (asterisk). Scale bars: 500 μm (**A**); 300 μm (**C, J, K**); 200 μm (**E, F**); 250 μm (**H, J**); 100 μm (**B**).

An important consequence of aging is the development of cancer [34]. Thus, a further confirmation of the premature aging of the K5-CYLD^C/S^ mice was the observation that transgenic animals (of both lines) develop spontaneous tumors of diverse origin when they reached about 8 months of age, while tumors were not detected in Control mice of similar age. The number of tumors originated in K5-CYLD^C/S^ mice is summarized (Table 1). We observed the development of skin tumors, such as a squamous cell carcinoma (Fig. 10A and B) and a hair follicle-derived tumor (trichofolliculoma) (Fig. 10C). Pulmonary adenocarcinomas (acinar ADC and papillary-predominant ADC) were also detected (Fig. 10D-F). A differentiated hepatocellular carcinoma (HCC) and a hepatocellular adenoma were also found in two transgenic mice (Fig. 10G and H). K5-CYLD^C/S^ mice also exhibited well differentiated gastric adenocarcinomas (Fig. 10J) and one *in situ* gastric carcinoma (Fig. 10K). Other tumor less frequently developed in K5-CYLD^C/S^ mice was a mammary adenomyoepithelioma (Fig. 10I). These findings indicate that the lack of the DUB function of CYLD makes transgenic mice more susceptible to the development of tumors, confirming *in vivo* the role of *Cyld* as a tumor suppressor gene in distinct organs.

**Table 1:** Number of tumors spontaneously developed in the K5-CYLD^^C/S^^ mice.

Genotype	Skin and HF Tumors	Lung ADC	Gastric ADC	Gastric Carcinoma *in situ*	HCC	Mammary ADC
Control	0/10	0/6	0/7	0/7	0/6	0/1
K5-CYLD^C/S^	2/10	4/6	3/8	1/8	2/6	1/2

Number of animals that have developed each type of tumor, as well as the number of mice that has been analyzed is shown. Mice of 8-29 months of age were analyzed.

**Figure 11:**
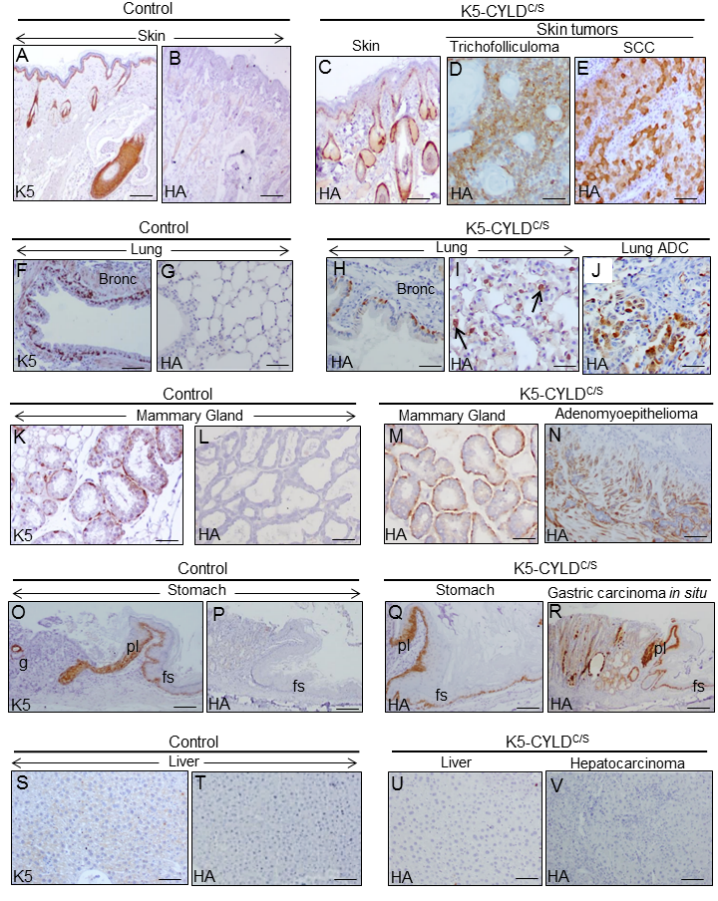
**Analysis of the expression of the transgene in the tumors developed in the K5-CYLD^C/S^ mice and in their matched non tumoral tissue.** Immunohistochemical staining with K5 and HA antibodies. (**A, B**) Snout sections from Control mice. K5 expression in the basal layer of the epidermis, HF and the immature cells of the sebaceous glands (**A**); HA is not detected (**B**). (**C)** HA expression in the snout of transgenic mice following the K5 expression pattern. HA expression in the tricofolliculoma of the snout (**D**) and in the SCC of the back skin of K5-CYLD^C/S^ mice (**E**). (**F-G**) K5 expression in the basal layer of the epithelium of bronchia and bronchioles of Control mice (**F**); no HA staining was observed (**G**). (**H, I**) HA in bronchia and bronchioles of transgenic mice (**H**) and in alveolar cells (**I**). (**J**) HA expression in the lung ADC. (**K, L**) K5 expression in the myoepithelial cells around the mammary secretory acini of Control mice (**K**); HA is not detected (**L**). (**M**) HA in the mammary secretory acini of lactating transgenic mice following the K5 expression patter. (**N**) HA expression in the mammary adenomyoepithelioma. (**O, P**) Stomach from a Control mice showing K5 expression in the aglandular epithelia (forestomach, fs), plica (pl), and in scattered glands (g) (**O**); HA is not expressed (**P**). (**Q**) Expression of HA in the stomach of transgenic mice following the K5 expression pattern. (**R**) Gastric carcinoma *in situ* expressing HA. (**S, T**) Neither K5 nor HA are expressed in the liver of Control mice. (**U**) HA is not detected in hepatocytes of K5-CYLD**^C/S^** mice. (**V**) HA is not expressed in the hepatocarcinomas (HCC) of transgenic animals. Scale bars: 300 μm (**A-C; N, R**); 150 μm (**D, E, I, J**); 70 μm (**K-M**); 250 μm (**G, O, P**); 200 μm (**F, H, Q, V**); 100 μm (**S-U**). ADC: adenocarcinoma.

Next, we performed immunostaining with the specific HA antibody to examine whether the transgene was expressed in these tumors; our data showed that the transgene was expressed in all tumors- as well as in the corresponding non tumoral tissues- except for the HCC, in which the transgene was not detected (Fig. 11).

To further reinforce our findings showing the role of CYLD as a protector from aging and tumor development, we analyzed the phenotype of the transgenic mice in a different genetic background (i.e., FVB/N background) and found that K5-CYLD^C/S^ /FVB/N mice develop similar alterations to those described above, i.e., they showed premature aging of skin and other organs (thymus, pancreas, stomach, lung etc.) (Fig. S10 and S11) and develop spontaneous tumors (from 8-10 months onwards), while their age-matched Control littermates do not show tumor development (Fig. S12).

### The reduction of NF-κB activity decreases the expression of TNF-α and p16 in keratinocytes

**Figure 12:**
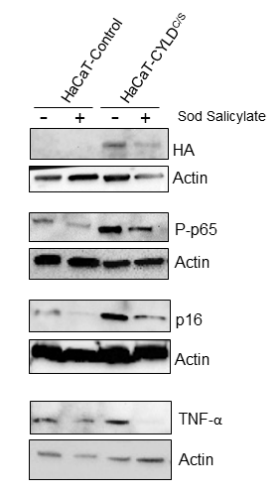
**The reduction of the NF-κB overactivation in keratinocytes expressing the CYLD^C/S^ mutant decreases the expression of the biomarker of aging p16 and TNFα.** HaCaT (Control and CYLD^C/S^) cells were treated with sodium salicylate for 48h when indicated (+). WB shows that HaCaT-CYLD^C/S^ cells exhibit increased levels of expression of P-p65, p16 and TNFα, but the treatment with sodium salicylate, which reduced P-p65 levels, also decreases p16 and TNF-α levels in these cells. Actin was used as a control loading.

To establish a causal relationship between CYLD lack of function, and NF-κB activation and aging, we studied the properties of the HaCaT keratinocytes expressing the CYLD^C/S^ mutant [6,9]. We checked that HaCaT-CYLD^C/S^ cells presented overactivation of the NF-κB pathway (Fig. 12) and increased levels of p16 expression, whose increment is considered a biomarker of aging [26]. However, treatment of HaCaT keratinocytes with sodium salicylate reduced NF-κB activation in both, Control and CYLD^C/S^ expressing cells, and reduced the overexpression of p16 found in the mutant HaCaT-CYLD^C/S^ keratinocytes to those levels showed in the HaCaT-Control cells (which contain a functional CYLD) (Fig. 12). In addition, we also found that NF-κB inhibition in the HaCaT-CYLD^C/S^ cells reduces the expression of TNF-α (target of NF-κB). Thus, our results in keratinocytes suggest a causal relationship between the lack of CYLD function, the NF-κB hyperactivation, and the expression of aging hallmarks and inflammatory cytokines.

## DISCUSSION

We have generated a new model of transgenic mice, the K5-CYLD^C/S^ mice, carrying the mutant CYLD^C/S^ construct [6] under the control of the keratin 5 (K5) promoter. The resultant CYLD^C/S^ mutant protein lacking the DUB function of CYLD, acts as a dominant negative of the endogenous CYLD. The study of our transgenic mice has allowed us to know that the DUB function of CYLD is essential for the maintenance of the homeostasis of the skin and other organs, including thymus, lung, stomach, etc. preventing the development of cancer. Notably, we have discovered a new function of CYLD as a suppressor of aging.

In the skin, we have found the importance of CYLD for the maintenance of hair follicle homeostasis and for the regulation of the hair growth cycle, showing that the DUB function of CYLD is essential for HF anagen induction of the second hair cycle. Moreover K5-CYLD^C/S^ mice show an early diffuse alopecia, progressive with age, which could be the result of both the constitutive activation of the canonical pathway of NF-κB, and the increased expression of its targets, the pro-inflammatory cytokines TNF-α and IL-6, as inflammation is known to be linked to the development of the most common form of alopecia in humans, the androgenetic alopecia [35]. The K5-CYLD^C/S^ transgenic mice show in addition hyperplastic and orphan sebaceous glands, reminiscent of those found in another model of transgenic mice, the *Cyld^E∆9/∆9^* mice, also deficient in its DUB function [36]. In both types of transgenic mice the increased c-Myc activation found in skin could be mediating disorders of the sebaceous glands, as c-Myc has been characterized as a key regulator of homeostasis of these glands [37].

In addition to the skin appendages changes, the K5-CYLD^C/S^ mice show epidermal alterations, mainly impaired keratinocyte differentiation, thus confirming *in vivo* the results that our group have previously described using a model of skin equivalents of human HaCaT keratinocytes [9], in which we demonstrated that the overexpression of the wild-type CYLD (CYLD^wt^) promoted keratinocyte differentiation, whereas the expression of the mutant CYLD^C/S^ prevented, through the activation of the JNK pathway, the epidermal differentiation [9]. Now our results *in vivo*, ratify the relevance of the DUB function of CYLD for epidermal differentiation, and also show the overactivation of JNK in the skin of transgenic mice.

A very relevant finding of our studies is that CYLD acts as a suppressor of aging, suggesting that CYLD is necessary to maintain the homeostasis of many organs, protecting them from premature aging. The anti-aging function of some well-known tumor suppressor genes (i.e. PTEN, Ink4/Arf) has been previously reported [16,38], but the role of CYLD as an aging protector is a function not previously described. Supporting our results, CYLD deficiency in *Drosophila melanogaster* shortened life expectancy [39]. In addition, we show that levels of CYLD diminished in the skin and thymus of aged mice, as well as in the mammary gland (Alameda et al, not published) and other group has described it decreased expression in aged lungs [40].

One of the first manifestations of the early aging of the K5-CYLD^C/S^ mice is progressive diffuse alopecia, an alteration usually found in other models of mice exhibiting premature aging [41], and in progeria human diseases, such as Werner syndrome and Hutchinson-Gilford progeria [42]. Likewise, progressive hair loss related to age is also a hallmark of aging in humans [43]; as well as decreased hair growth [41], which we also find in our transgenic mice. Other symptoms of aging in both mice and humans are aging of the skin, mainly characterized by epidermal atrophy [44]; loss of panniculus adipose [45] and sebaceous gland hyperplasia [46]. We have observed all these signs of aging in the skin of young K5-CYLD^C/S^ mice (from 3 to 5 months of age), but they were not found in control mice of the same age; in fact these aging features are characteristics of 24-month-old control mice. Additionally the K5-CYLD^C/S^ mice show increased levels of both p16 and p19 in the skin, having been widely accepted that the expression of these proteins is augmented in aged tissues in both mice and humans [26,27]. Also, the K5-CYLD^C/S^ mice show increased levels of γH2AX, which is also considered a marker of aging [28]. Therefore, all these changes found in the skin of our transgenic mice lacking a functional CYLD suggest a role for CYLD as a protector of skin aging.

Chronic activation of NF-κB in the skin appears as the key alteration causing the premature aging of this organ in K5-CYLD^C/S^ mice, as it has been described that the main mechanism responsible for both physiological and pathological aging is the activation of NF-κB [18]. Accordingly, transgenic mice deficient in the p50 NF-κB subunit also show hyperactivation of the classical pathway of NF-κB and accelerated aging [47]. Although the skin of K5-CYLD^C/S^ mice show hyperactivation of other molecules, that also may favor aging (as JNK, Akt and c-Myc), it is interesting to note that the mechanism proposed through which the activation of these other pathways promotes aging is by activating NF-κB in turn [4].

Besides the skin, other organs present accelerated aging in our transgenic mice, among them, the early aging of the thymus is very remarkable, appearing also in this case the NF-κB over-activation as the likely mechanism causing the aging, as it has been previously reported that NF-κB is critically involved in the aging of the thymus [19,32]. The premature aging of other organs of the K5-CYLD^C/S^ mice could be the result of the early thymic involution (since it is considered one of the main regulators of physiological aging) as well as to the overexpression of TNF-α and IL-6, as it is known that these proinflammatory cytokines can affect distant organs causing systemic inflammation, compromising the homeostasis of the tissues and in turn promoting the accelerated aging [22]. Accordingly, elevated expression of TNF-α and IL-6 by the over-activation of NF-κB has been proven to occur in both physiological aging and in progeria human diseases [48]. Our transgenic mice show inflammation and early aging in numerous organs (lung, stomach etc.), which could be due either to a systemic effect of TNF-α and IL-6 in the skin and/or to the expression of the transgene in these organs. These possibilities will be analyzed.

It is interesting that although there are two other models of transgenic mice that, similar to the K5-CYLD^C/S^ mice, express a mutated form of CYLD that causes the lack of DUB activity in the epidermis and other tissues (the CYLD^m^ [7] and the CYLD^EΔ9/Δ9^ mice [36]); however, no premature aging has been described in neither of these two models. In the CYLD^m^ mice it was demonstrated the activation of JNK/AP1 in keratinocytes and skin tumors, though no significant increase in NF-κB activation was detected. Activation of c-Myc was observed in the epidermis of the CYLD^EΔ9/Δ9^ mice, and no reference to NF-κB overactivation is mentioned. Thus, it seems that the most likely reason for these other mice to age normally may be the absence of NF-κB hiperactivation. It also suggests that although both JNK and c-Myc activation might contribute to aging, the most important pro-aging pathway is that of NF-κB, as it has been widely demonstrated before [18].

A relevant phenotype of the K5-CYLD^C/S^ mice is also their susceptibility to spontaneously develop different types of tumors from an early age (8 months). Among them it has been shown the growth of skin tumors, which are likely the result of the molecular alterations observed in the skin of the transgenic mice, i.e. the overactivation of NF-κB, JNK, c-Myc and Akt. Different evidences support the role of these proteins in skin cancer development and progression, i.e., the activity of NF-κB increases with the skin tumor progression, supporting the pro-tumoral action of NF-κB in the cutaneous SCC [49]; also, NF-κB and JNK activation cause tumor development in familial cylindromatosis patients [1,2]. The amplification or deregulation of c-Myc causes the genesis and tumor promotion of cutaneous SCC, and we have described the increased malignancy of cutaneous SCCs overexpressing c-Myc [50]. Our group has also found that the constitutive activation of Akt in the basal keratinocytes of the K5-myrAkt transgenic mice promotes the development and malignancy of cutaneous SCC [51].

But in addition to the skin tumors, other tumor types are found with a high frequency in the K5-CYLD^C/S^ mice, such as those developed in the lung, liver, and stomach. It is known that in addition to keratinocytes, the K5 regulatory elements direct the expression of the transgene to other cell types that express K5, including those in the mammary gland, stomach and lung [24]. Thus, the growth of tumors in these organs may be the direct consequence of the expression of the transgene. In fact, the lack of CYLD function has been detected in many human tumor cells of lung, stomach and breast cancer, in which the activation of NF-κB, JNK and/or c-Myc appears as the mechanisms through which CYLD downregulation promotes tumor development [10–12]. But, in addition, K5-CYLD^C/S^ mice form tumors derived from cells that do not express the transgenic protein, such as the hepatocytes. In these cases, tumors may develop as a consequence of the premature aging of the transgenic mice. In this context, it is remarkable that all types of tumors arise mainly in organs in which premature aging and inflammation has been noticed, such as in the skin, stomach, lung and liver. Therefore, it seems that tumor development could be the consequence of these pathologies, as the relationship between aging, chronic inflammation and tumor development is well established [34,52].

Thereby, the study of the K5-CYLD^C/S^ mice demonstrates the essential role of CYLD, *in vivo*, as a tumor suppressor of wide spectrum, providing an excellent model for studying, *in vivo*, the signaling pathways throughout CYLD exerts its tumor suppressor role in different types of cancer. In addition, our results suggest that the role of CYLD as an aging suppressor may be a mechanism through which CYLD acts as a tumor suppressor. In support of our hypothesis is the fact that although the function of CYLD as a tumor suppressor has been reported for different types of cancer [53]; however, none of the other two models of mutant CYLD transgenic mice (i.e. the CYLD^m^ and *Cyld^E∆9/∆9^* mice) develop cancer spontaneously, strongly suggesting that the reason for this may be that they do not age prematurely. Therefore, the K5-CYLD^C/S^ mice constitute a suitable *in vivo* model for the study of the mechanisms through which NF-κB activation promotes aging, and to test putative targets aimed to delay the devastating effects of progeria syndromes. Moreover, the K5-CYLD^C/S^ mice offer a useful model for the study of the mechanisms involved in the chronological aging of the human skin, as it recapitulates its fundamental alterations.

## MATERIALS AND METHODS

### Generation of transgenic mice

HA-tagged murine CYLD^C/S^ [6] was placed under the control of a 5.2 kb 5’-upstream fragment of bovine K5 promoter and a rabbit β-globin intron (Figure 1A). Transgenic mice were generated by microinjection of this construct into B6D2F2 embryos using standard techniques. Mice were genotyped by PCR analysis of tail genomic DNA using primers specific for the rabbit β-globin intron. Wild type non-transgenic littermates were used as control animals. Two lines of transgenic mice were established (K5-CYLD^C/S^-A and K5-CYLD^C/S^-X). In addition, transgenic K5-CYLD^C/S^-X mice were derived to FVB/N genetic background and analyzed.

### Induced adult hair cycle

The hair parallel to the paravertebral line on the back skin of 7-week-old control and transgenic mice were removed. This procedure leads to synchronized development of anagen hair follicles. Tissues were obtained at days 16 and 21 after depilation and fixed in 10% buffered formalin. The distinct phases of hair follicle development were determined as previously described [5]. Body weight of the two groups of mice was not significantly different at any point of time during the experiment. Six animals of each genotype were analyzed.

### BrdU labeling

Mice received an intraperitoneal injection of BrdU 120 mg/kg body weight 1 h before sample harvesting. BrdU incorporation was detected by immunohistochemistry of paraffin-embedded sections using an anti-BrdU monoclonal antibody (Roche).

### Isolation of thymic cells

Thymus from 1year old mice were collected and immersed in PBS, then mechanical disaggregation with syringe and filter system was performed.

### Ethics statement

All animal experimental procedures were performed according to European and Spanish laws and regulations (2007/526/CE) and approved by the Ethics Committee for Animal Welfare of CIEMAT and by the legal authority (protocol code PROEX182/15).

### Histology and immunohistochemistry

Mouse tissues were dissected and fixed in 10% buffered formalin or 70% ethanol and embedded in paraffin. Five μm-thick sections were used for H&E staining or immunohistochemical preparations. Antibodies used in immunostaining were: antibodies against HA (3724, Cell Signaling Technology); CYLD (SAB4200061), Involucrin (I9018) and Sma (C-6198) (Sigma-Aldrich); K5, K10, Filaggrin and Loricrin (Covance).

### Immunoblots and immunoprecipitation

Antibodies used in Western blots were: Actin (sc-1616), GAPDH (sc-25778), IκBα (sc-371), p65 (sc-8008), Bcl-3 (sc-185) and P-c-Myc (sc-8000) (Santa Cruz Biotechnology); HA (3724), Ubiquitin (3936), P-Akt (4068), P-IκBα (2859), P-JNK (4668) and P-p65 (3033) (Cell Signaling Technology); IL-6 (9324) (R&D Systems); CYLD (SAB4200061, Sigma-Aldrich); c-Myc (626802, Biolegend); TNF-α (654250, Calbiochem); IKKγ (IMG-5480-2, Novus Biologicals); K63-Ubiquitin (ab179434), p19 (ab80) and p16 (ab51243) (Abcam) and γH2AX (05-636) (Millipore). For immunoprecipitation 300 µg cell lysate were incubated at 4ºC overnight. Then washed, and performed the immunoblotting.

### Determination of TNF-α and IL-6 in serum

Mice were anesthetized and blood were obtained (300µl) by puncture of the vein of the tail. Serum was stored at -20ºC until assay. Serum from Control (n=16) and K5-CYLD^C/S^ (n=16) mice of 15-20 months of age were analyzed for the expression of both TNF-α and IL-6 cytokines (LEGENDplex Multi-Analyte Flow Assays Kit, Biolegend). Cytokines were measured in a Tecan GENios Microplate Reader (Tecan Trading AG, Switzerland).

### Cell culture and treatment

HaCaT cell lines of human keratinocytes were cultured in DMEM supplemented with 10% fetal calf serum. HaCaT-Control, HaCaT-CYLD^C/S^ cells have been previously described [54]; briefly, HaCaT-CYLD^C/S^ cells were transfected in a stable manner with the β-Actin-CYLD^C/S^ construct and are deficient in the DUB function of CYLD. Cells were grown in the presence of G418 (0.4mg/ml). When indicated, cells were incubated with 10mM sodium salicylate (S3007, Sigma-Aldrich) in DMEM for 48h.

## SUPPLEMENTARY MATERIAL

Supplementary File
