# Supplementary material for: Premature aging and cancer development in transgenic mice lacking functional CYLD
Source: Aging (Albany NY). 2019 Jan 10;11(1):127–59. doi: 10.18632/aging.101732 (PMC6339805; doi:10.18632/aging.101732)
Supplement: Supplementary File [file aging-11-101732-s001.pdf]

SUPPLEMENTARY MATERIAL

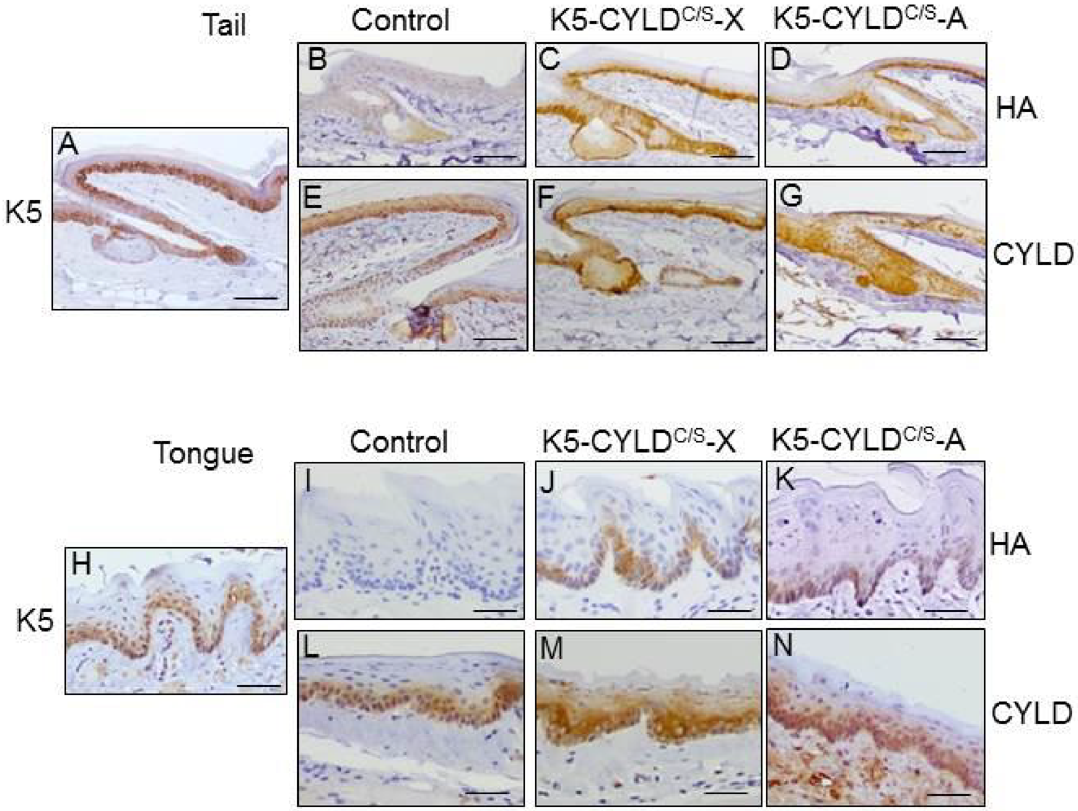

Figure S1. Analysis of the transgene expression in tail skin and tongue. (**A**) Representative staining of K5 in the skin of a control mouse. (**B-D**) HA and (**E-G**) CYLD expression in the tail skin of Control and transgenic mice. Observe that HA is not detected in Control sections (**B**), while HA expression in the tail of K5-CYLD$^{C/S}$ mice follows the K5 expression pattern (**C-D**). (**H**) Representative sections showing the expression of K5 in the tongue of a control mouse. (**I-K**) HA and (l-n) CYLD expression in the tongue of Control and transgenic mice. HA is not expressed in the tongue of Control mice (**I**), while HA expression in the tongue of transgenic mice follows the K5 expression pattern in control mice (**J, K**). Sections correspond to 1-month-old mice. Scale bars: (**A-D**) 220 µm; (**E-G**) 200 µm; (**H, J, K**); 180 µm; (**I, L-N**) 160 µm.

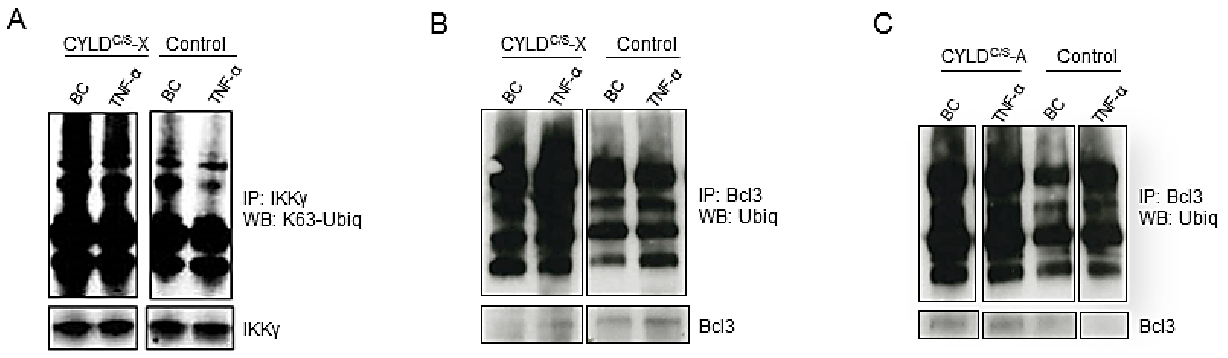

Figure S2. Lack of CYLD DUB function in the K5-CYLD$^{C/S}$ mice. Functional analysis of the CYLD$^{C/S}$ transgene, by IP of back skin protein extracts with an IKKγ or Bcl3 specific antibodies, in the presence and absence of TNF-α. Western blots using Ubiquitin and IKKγ specific antibodies are shown. (**A**) Observe the lack of DUB function in transgenic mice checked as elevated levels of polyubiquitinated IKKγ in both the basal contition (BC) and after TNF-α treatment. (**B, C**) Observe the increased levels of ubiquitinated Bcl3 in the back skin of transgenic mice in both states: without TNF-α stimulation (BC, basal condition), and after TNF-α treatment. Similar results were obtained for both K5-CYLD$^{C/S}$-X (**B**) and K5-CYLD$^{C/S}$-A (**C**) mice. BC: basal condition, i.e., without TNF-α stimulation. (**A, B**) when indicated, cells were incubated for 15 min with TNF-α treatment; (**C**) when indicated cells were incubated 40 min with TNF-α.

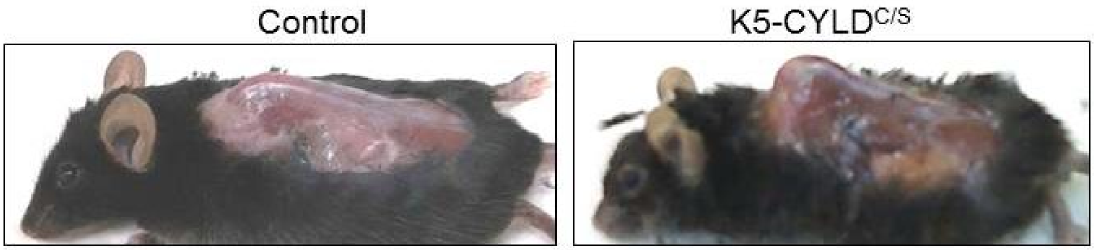

Figure S3. External phenotype of the K5-CYLD$^{C/S}$ mice. Representative image showing kyphosis in the back of a transgenic mouse. Both Control and K5-CYLD$^{C/S}$ mice are littermates of 11 months-old.

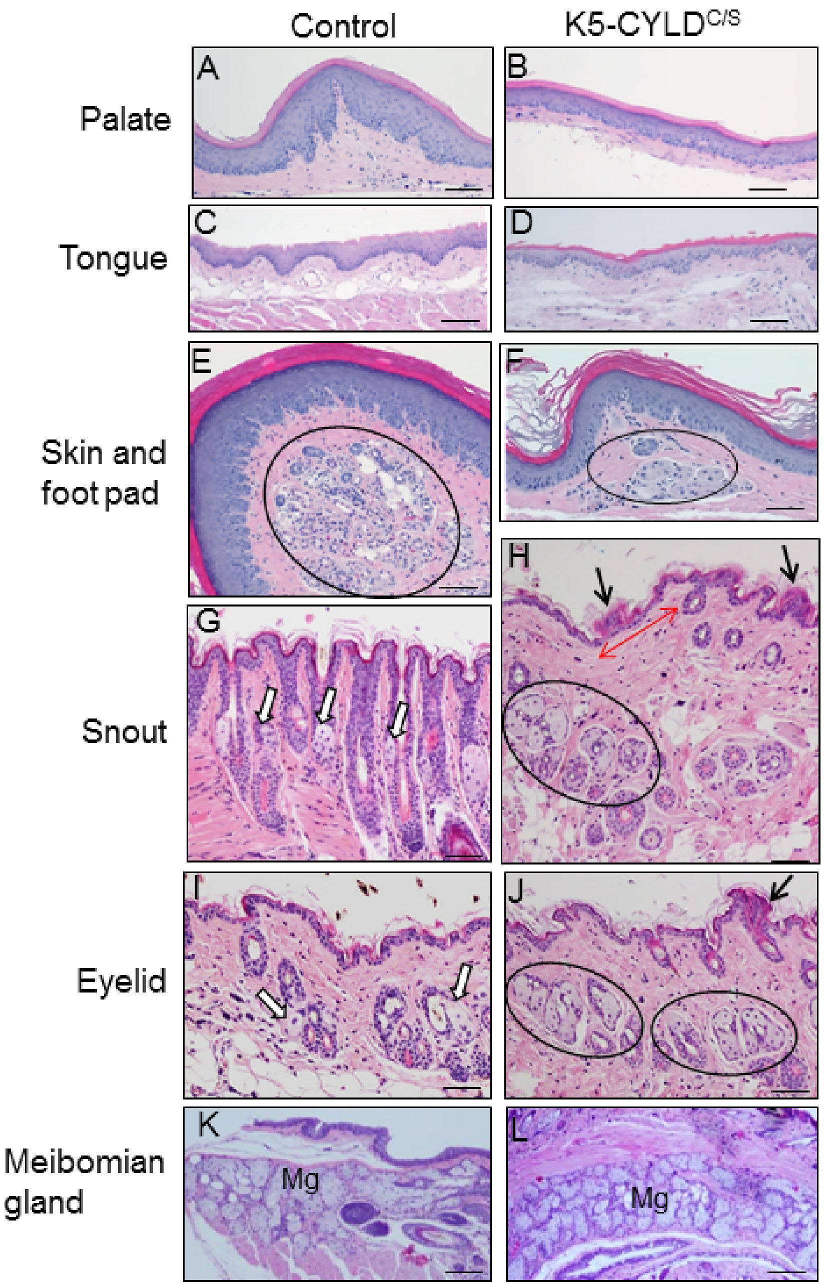

Figure S4. Histological analysis showing the premature aging of different epithelia of the K5-CYLD$^{C/S}$ mice. Sections from both Control (**A, C, E, G, I, K**) and transgenic mice (**B, D, F, H, J, L**) of 5- (**G-J**) and 20-month-old are shown. (**A, B**) Palate epithelium; note the atrophy in that of K5-CYLD$^{C/S}$ mice (**B**). (**C, D**) Stratified epithelium of the tongue. Note the thinning of the epithelium in transgenic mice (**D**). (**E, F**) Scarce eccrine glands in the hind limb foot pads of the K5-CYLD$^{C/S}$ mice are appreciated (circle). (**G, H**) Snout skin of 5-month-old mice. Highly abundant hyperplastic sebaceous glands are observed in the transgenic mice (circles in **H**); additionally, an atrophic epidermal area (double-headed red arrow) and epidermal ridges (black arrows) are shown. Eyelid skin of 5-month-old mice (**I, J**); note the presence of numerous hyperplastic sebaceous glands in the K5-CYLD$^{C/S}$ mice (enclosed in circles), in clear contrast with those found in the corresponding tissue of Control mice (**I**, white arrows). Also note the absence adipose tissue in the eyelid transgenic mice and the presence of epidermal ridges (**J**, black arrows). (**K, L**) Note the abundant hyperplastic Meibomian glands (Mg) found in the eyelids of K5-CYLD$^{C/S}$ mice (**L**) compared to those in Control mice (**K**). Images from 20-month-old mice are showed, although similar alterations are found in young transgenic mice (from 3 months-old). Scale bars: 250 µm (**A-F, K, L**); 150 µm (**G, H**) 230 µm (**I, J**).

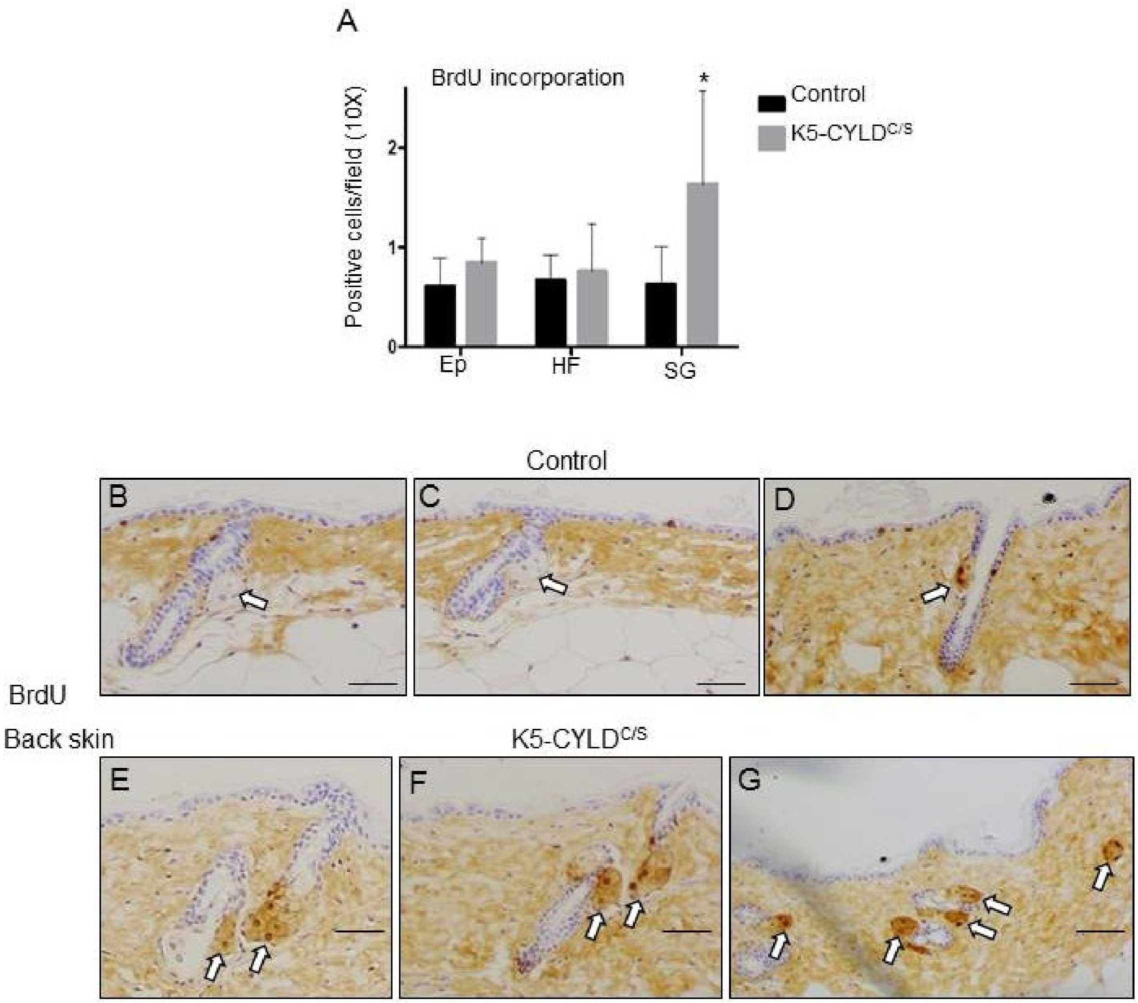

Figure S5. BrdU analysis in the back skin of Control and K5-CYLD$^{C/S}$ mice. (**A**) BrdU incorporation in the back skin of 1-year-old mice. Note the increased proliferation of the sebaceous glands from transgenic mice (4 Control and 5 transgenic mice were analyzed; error bars represent SEM; *P* value by Bonferroni multiple comparisons test: two-way ANOVA). P<0.05. (**B-G**) Representative image showing the BrdU staining in Control (**B-D**) and transgenic (**E-G**) sections of back skin. Observe the increased BrdU incorporation in the sebaceous glands of the K5-CYLD$^{C/S}$ mice. Scale bars: 150 µm. Ep: epidermis; HF: hair follicles; SG: sebaceous glands.

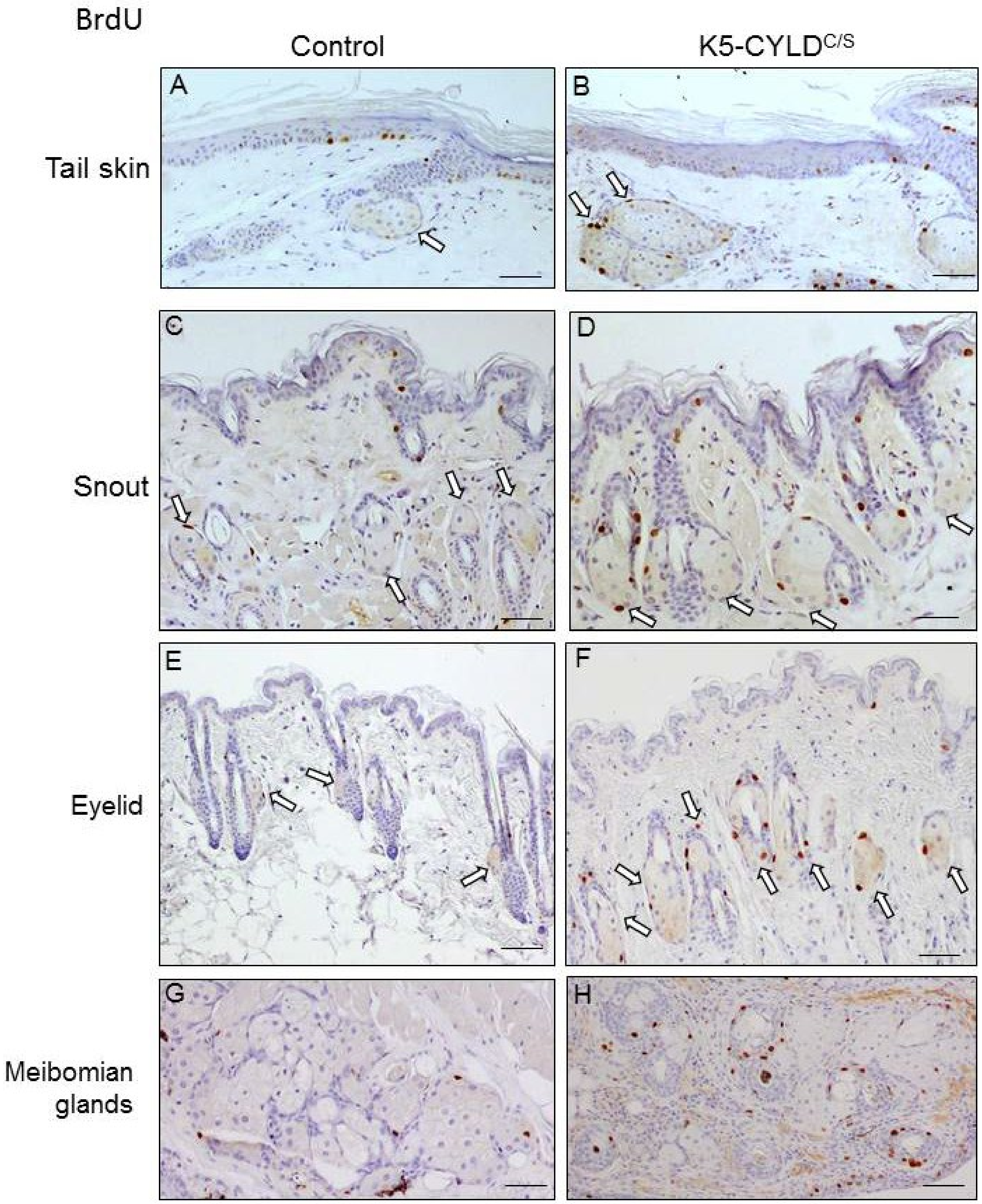

Figure S6. BrdU incorporation in the skin of Control and K5-CYLD$^{C/S}$ mice. BrdU staining in Control (**A, C, E, G**) and transgenic (**B, D, F, H**) sections showing increased signal in the hyperplastic sebaceous glands of the tail, snout and eyelid skin of the K5-CYLD$^{C/S}$ mice (**B, D, F**); as well as in the Meibomian glands (**H**) of the transgenic mice. White arrows point to sebaceous glands. Scale bars: 140 µm (**A-F**); 110 µm (**G, H**).

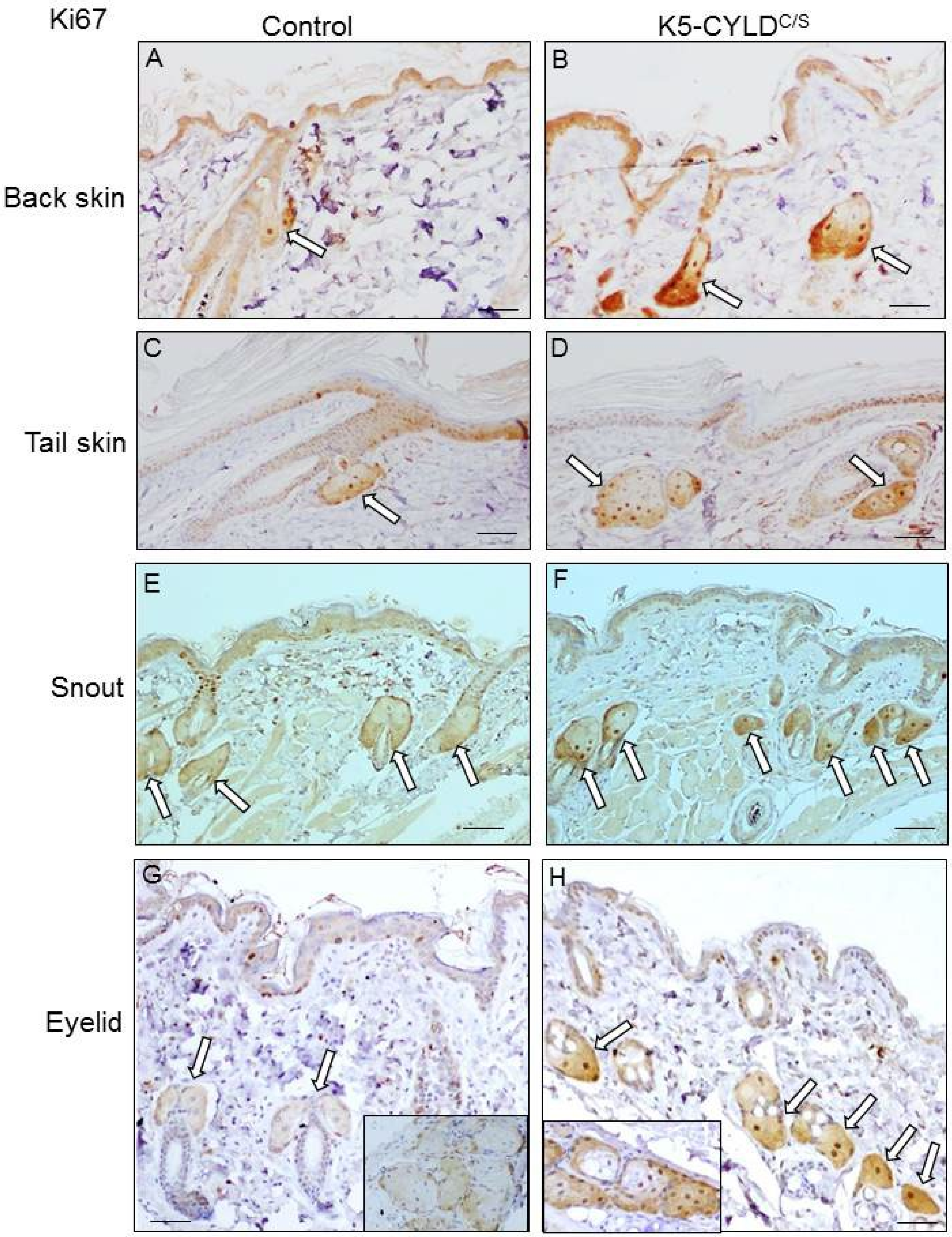

Figure S7. Analysis of cell proliferation in the skin of Control and K5-CYLD$^{C/S}$ mice. Ki67 staining in Control (**A, C, E, G**) and transgenic (**B, D, F, H**) sections showing increased signal in the hyperplastic sebaceous glands of the back, tail, snout and eyelid skin of transgenic mice. Also note that Meibomian glands of the K5-CYLD$^{C/S}$ mice are more proliferative than those of Control mice (compare insets in **G** and **H**). White arrows point to sebaceous glands. Scale bars: 160 µm (**A-F**); 120 µm (**G, H**).

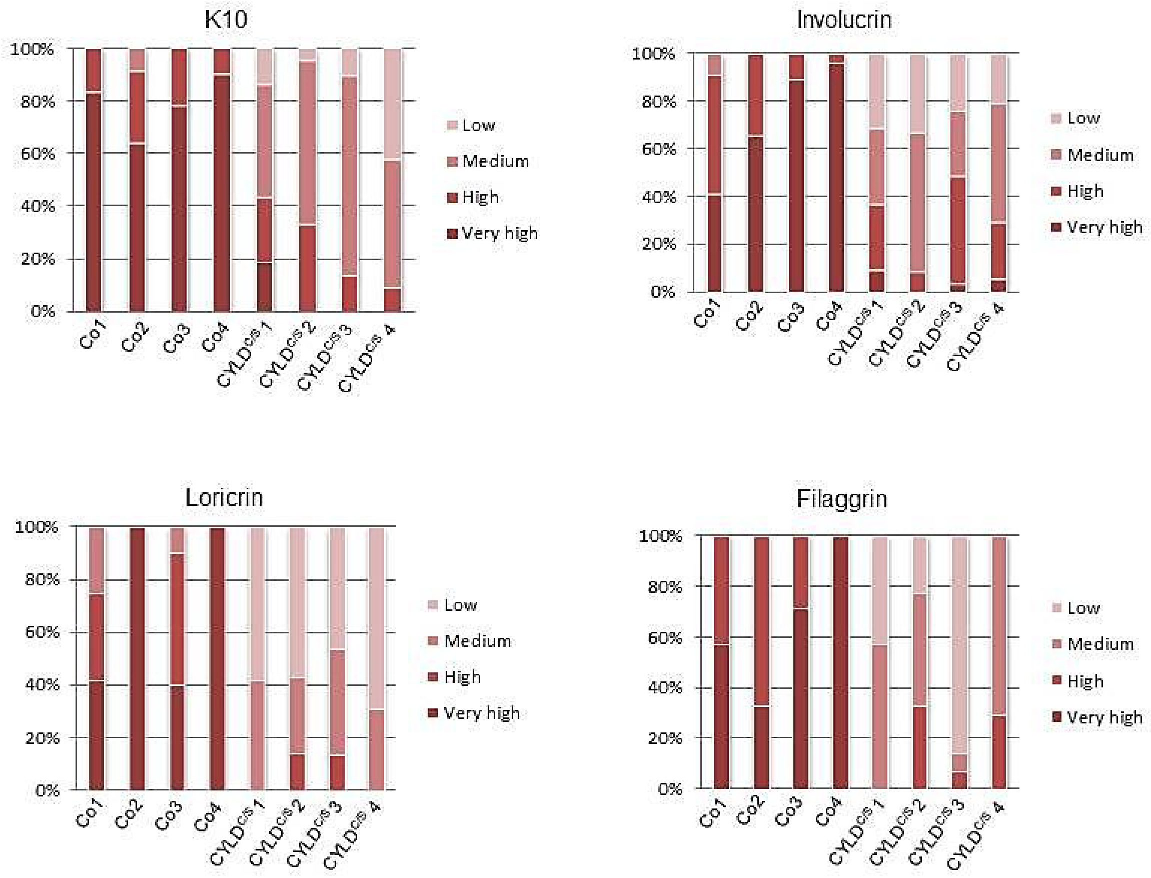

Figure S8. Semiquantitative analysis of the intensity of the expression of epidermal differentiation markers in Control and K5-CYLD$^{C/S}$ mice. 10-15 fields, 10X magnification, corresponding to K10, Involucrin, Loricrin and Filaggrin immunostainings were analyzed and quantified as very high, high, medium or low expression. Analysis of the expression in 4 animals per genotype and staining is showed. Co: Control animals; CYLD$^{C/S}$: K5-CYLD$^{C/S}$ transgenic mice.

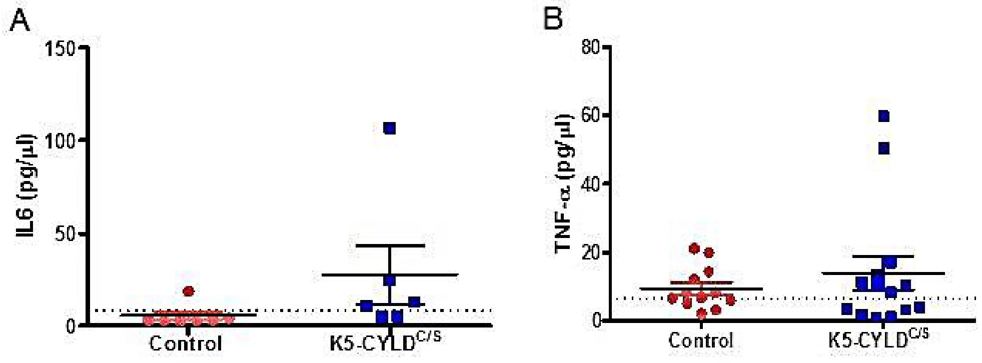

Figure S9. Increased levels of IL6 and TNF-α in the serum of K5-CYLD$^{C/S}$ mice. IL6 (**A**) and TNF-α (**B**) serum levels from 8 Control and 8 transgenic mice of both 16 and 22 months-old (i.e., a total of 16 control and 16 transgenic animals) were analyzed. Results show increased levels of both cytokines in the serum of transgenic mice, mainly of IL-6.

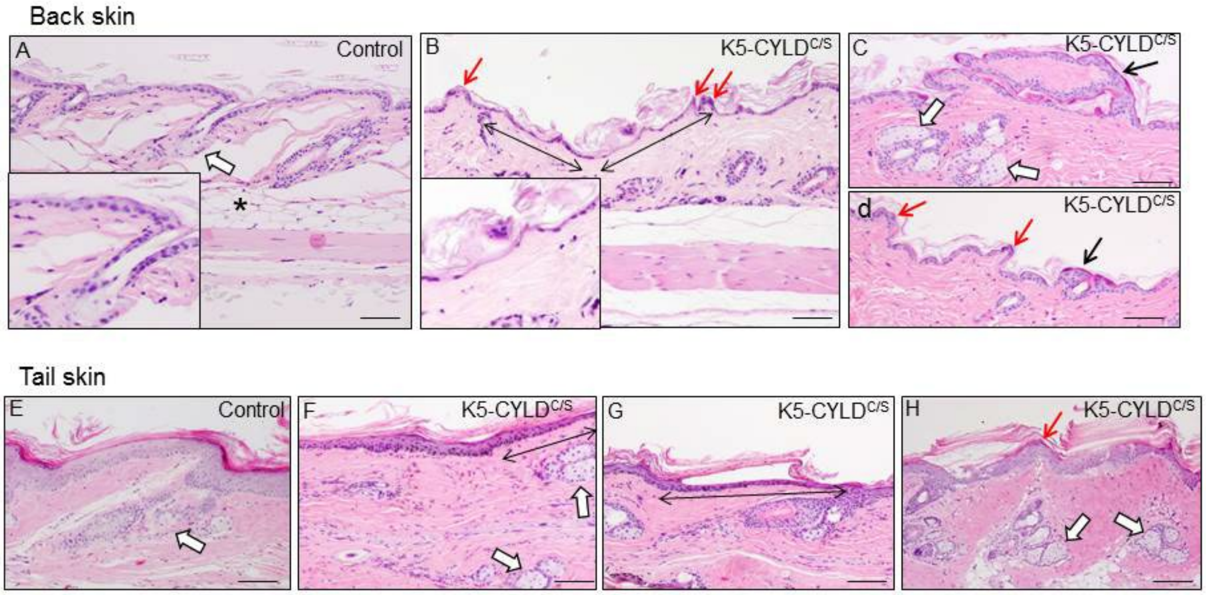

Figure S10. Phenotypic alterations in the skin of FVB/N-K5-CYLD$^{C/S}$ mice indicating that premature aging signs are independent of their genetic background. Representative images showing the main histological alterations presented by K5-CYLD$^{C/S}$ mice developed in a FVB/N genetic background (these are coincident with that observed in B6D2-transgenic mice). (**A-D**) Back skin sections from Control (**A**) and FVB/N-transgenic mice (**B-D**); (**E-H**) tail skin images from Control (**E**) and FVB/N-transgenic (**F-H**) mice. Observe areas of atrophy in the epidermis of the FVB/N-K5-CYLD$^{C/S}$ mice (double-headed arrows in **B,F,G**; compare inserts in **A,B**); epidermal ridges of pyknotic keratinocytes (red arrows in **B,D,H**); papilomatous hyperplasia (black arrows in **C, D**); hyperplasic and orphan sebaceous glands (white arrows in **C,F,H**) compared with normal-size sebaceous glands in Control (white arrows in **A, E**). Reduced number of hair follicles (**C, F, H**), and scarce adipose tissue (**B**) is observed in FVB/N-transgenic mice; asterisk in (**A**) indicates fat tissue in Control mice hypodermis. Five animals of each genotype (18-20-month-old) were analyzed. Scale bars: 250 µm (**A-D**); 300 µm (**E-H**).

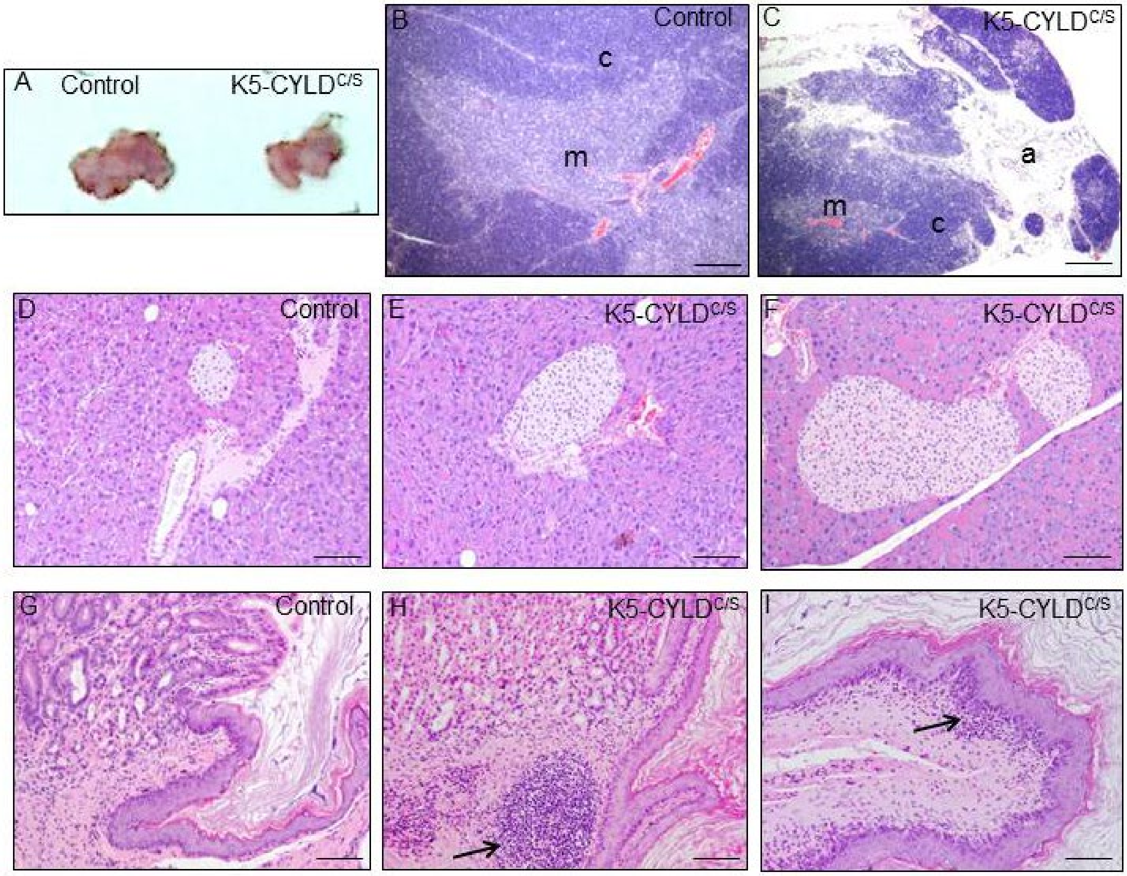

Figure S11. Signs of premature aging in the thymus, pancreas and stomach of FVB/N-K5-CYLD$^{C/S}$ mice. (**A**) Representative images showing the smaller size of the thymus of 3-month-old FVB/N-transgenic mice. (**B, C**) Thymic atrophy and infiltration of white adipose tissue (**A**) in the thymus of FVB/N-transgenic mice (**C**) compared to thymus of age-matched Control mice (b). Histopathologic analysis of pancreas (**D-F**) and stomach (**G-I**) from 18-month-old Control (**D, G**) and FVB/N-transgenic (**E, F, H, I**) mice. Note the hyperplasia of the Islets of Langerhans in the FVB/N-K5-CYLD$^{C/S}$ mice (**E, F**). (**H, I**) Representative images showing foci of inflammation in the stomach of FVB/N-transgenic mice (black arrows). Scale bars: 400 µm (**B, C**); 200 µm (**D-F**); 250 µm (**G-I**).

A **FVB/N-K5-CYLD$^{C/S}$ mice develop spontaneous tumors**

FVB/N Mouse genotype	Lung ADC	Mammary ADC
**Control**	0/5	0/3*
**K5-CYLD$^{C/S}$-X**	2/5	1/3*

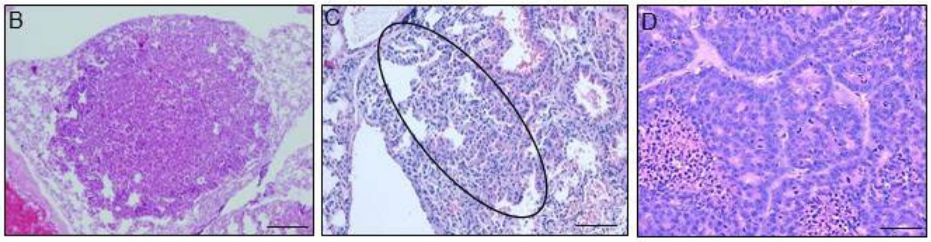

Figure S12. K5-CYLD$^{C/S}$ mice develop spontaneous tumors independently of their genetic background. Control and transgenic mice (FVB/N background) were analyzed (3 females and 2 males). (**A**) Lung and mammary adenocarcinomas were detected in aging FVB/N-K5-CYLD$^{C/S}$ mice. Number of animals that have developed each type of tumor, as well as the number of mice that have been analyzed is shown. (**B**) Image of lepidic lung adenocarcinoma developed in a FVB/-N-transgenic mouse. (**C**) Atypical adenomatous hyperplasia of lung in a FVB/N-K5-CYLD$^{C/S}$ mouse (circle). (**D**) Mammary adenocarcinoma of high grade in a female FVB/N-transgenic mouse. ADC: Adenocarcinoma; (*): female mice. Scale bars: 500 µm (**A**); 150 µm (**B, C**).

Table S1. Number of mice whose skin has been analyzed.

Genotype	Age (months)							
	1 m	3 m	5 m	8 m	12m	20 m	21-24 m	25-30 m
**Control**	4	6	5	4	4	7	4	8
**K5-CYLD$^{C/S}$-X**	4	6	5	4	5	10	3	9

The number of animals analyzed at the indicated months of age is showed.

Table S2: Number of mice whose thymus has been analyzed.

Genotype	Age (months)			
	1m	2.5 m	3m	3.5m
**Control**	3	3	8	3
**K5-CYLD$^{C/S}$-X**	3	3	8	3

The number of animals analyzed at the indicated months of age is showed.
